# Four years of climate warming reduced dark carbon fixation in coastal wetlands

**DOI:** 10.1093/ismejo/wrae138

**Published:** 2024-07-25

**Authors:** Bolin Liu, Lin Qi, Yanling Zheng, Chao Zhang, Jie Zhou, Zhirui An, Bin Wang, Zhuke Lin, Cheng Yao, Yixuan Wang, Guoyu Yin, Hongpo Dong, Xiaofei Li, Xia Liang, Ping Han, Min Liu, Guosen Zhang, Ying Cui, Lijun Hou

**Affiliations:** Key Laboratory of Geographic Information Science of the Ministry of Education, School of Geographic Sciences, East China Normal University, 500 Dongchuan Road, Shanghai 200241, China; State Key Laboratory of Estuarine and Coastal Research, Yangtze Delta Estuarine Wetland Ecosystem Observation and Research Station, East China Normal University, 500 Dongchuan Road, Shanghai 200241, China; Key Laboratory of Geographic Information Science of the Ministry of Education, School of Geographic Sciences, East China Normal University, 500 Dongchuan Road, Shanghai 200241, China; Key Laboratory of Geographic Information Science of the Ministry of Education, School of Geographic Sciences, East China Normal University, 500 Dongchuan Road, Shanghai 200241, China; State Key Laboratory of Estuarine and Coastal Research, Yangtze Delta Estuarine Wetland Ecosystem Observation and Research Station, East China Normal University, 500 Dongchuan Road, Shanghai 200241, China; Key Laboratory of Geographic Information Science of the Ministry of Education, School of Geographic Sciences, East China Normal University, 500 Dongchuan Road, Shanghai 200241, China; State Key Laboratory of Estuarine and Coastal Research, Yangtze Delta Estuarine Wetland Ecosystem Observation and Research Station, East China Normal University, 500 Dongchuan Road, Shanghai 200241, China; State Key Laboratory of Estuarine and Coastal Research, Yangtze Delta Estuarine Wetland Ecosystem Observation and Research Station, East China Normal University, 500 Dongchuan Road, Shanghai 200241, China; State Key Laboratory of Estuarine and Coastal Research, Yangtze Delta Estuarine Wetland Ecosystem Observation and Research Station, East China Normal University, 500 Dongchuan Road, Shanghai 200241, China; Key Laboratory of Geographic Information Science of the Ministry of Education, School of Geographic Sciences, East China Normal University, 500 Dongchuan Road, Shanghai 200241, China; Key Laboratory of Geographic Information Science of the Ministry of Education, School of Geographic Sciences, East China Normal University, 500 Dongchuan Road, Shanghai 200241, China; Key Laboratory of Geographic Information Science of the Ministry of Education, School of Geographic Sciences, East China Normal University, 500 Dongchuan Road, Shanghai 200241, China; Key Laboratory of Geographic Information Science of the Ministry of Education, School of Geographic Sciences, East China Normal University, 500 Dongchuan Road, Shanghai 200241, China; State Key Laboratory of Estuarine and Coastal Research, Yangtze Delta Estuarine Wetland Ecosystem Observation and Research Station, East China Normal University, 500 Dongchuan Road, Shanghai 200241, China; State Key Laboratory of Estuarine and Coastal Research, Yangtze Delta Estuarine Wetland Ecosystem Observation and Research Station, East China Normal University, 500 Dongchuan Road, Shanghai 200241, China; State Key Laboratory of Estuarine and Coastal Research, Yangtze Delta Estuarine Wetland Ecosystem Observation and Research Station, East China Normal University, 500 Dongchuan Road, Shanghai 200241, China; Key Laboratory of Geographic Information Science of the Ministry of Education, School of Geographic Sciences, East China Normal University, 500 Dongchuan Road, Shanghai 200241, China; Key Laboratory of Geographic Information Science of the Ministry of Education, School of Geographic Sciences, East China Normal University, 500 Dongchuan Road, Shanghai 200241, China; State Key Laboratory of Estuarine and Coastal Research, Yangtze Delta Estuarine Wetland Ecosystem Observation and Research Station, East China Normal University, 500 Dongchuan Road, Shanghai 200241, China; State Key Laboratory of Estuarine and Coastal Research, Yangtze Delta Estuarine Wetland Ecosystem Observation and Research Station, East China Normal University, 500 Dongchuan Road, Shanghai 200241, China; State Key Laboratory of Estuarine and Coastal Research, Yangtze Delta Estuarine Wetland Ecosystem Observation and Research Station, East China Normal University, 500 Dongchuan Road, Shanghai 200241, China

**Keywords:** warming, dark carbon fixation, coastal wetlands, chemoautotrophy, carbon fixation pathway, metagenomics

## Abstract

Dark carbon fixation (DCF), conducted mainly by chemoautotrophs, contributes greatly to primary production and the global carbon budget. Understanding the response of DCF process to climate warming in coastal wetlands is of great significance for model optimization and climate change prediction. Here, based on a 4-yr field warming experiment (average annual temperature increase of 1.5°C), DCF rates were observed to be significantly inhibited by warming in coastal wetlands (average annual DCF decline of 21.6%, and estimated annual loss of 0.08–1.5 Tg C yr^−1^ in global coastal marshes), thus causing a positive climate feedback. Under climate warming, chemoautotrophic microbial abundance and biodiversity, which were jointly affected by environmental changes such as soil organic carbon and water content, were recognized as significant drivers directly affecting DCF rates. Metagenomic analysis further revealed that climate warming may alter the pattern of DCF carbon sequestration pathways in coastal wetlands, increasing the relative importance of the 3-hydroxypropionate/4-hydroxybutyrate cycle, whereas the relative importance of the dominant chemoautotrophic carbon fixation pathways (Calvin–Benson–Bassham cycle and W-L pathway) may decrease due to warming stress. Collectively, our work uncovers the feedback mechanism of microbially mediated DCF to climate warming in coastal wetlands, and emphasizes a decrease in carbon sequestration through DCF activities in this globally important ecosystem under a warming climate.

## Graphical Abstract

**Graphical Abstract:**
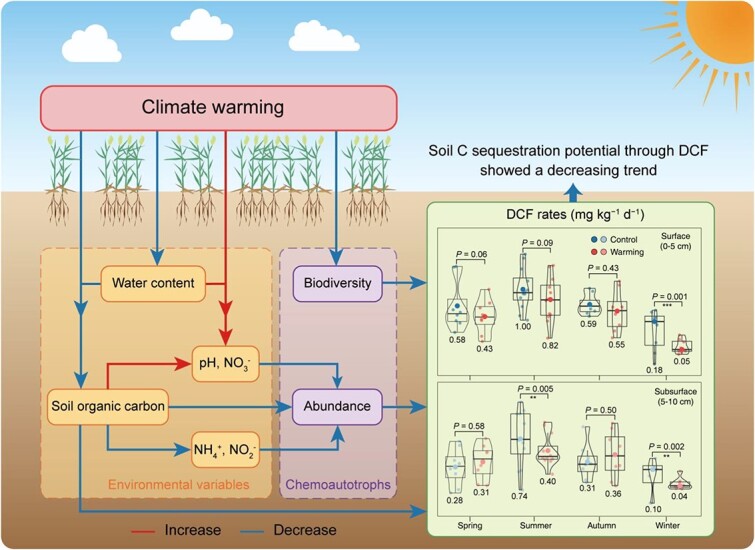

## Introduction

Coastal wetlands, located in the buffer zone between land and sea, are huge and long-term sustainable carbon sinks with high primary productivity but slow rates of organic matter decomposition [1]. This “blue carbon” ecosystem has emerged as one of the “nature-based” solutions in coping with global climate change [2]. Along with the well-known photosynthetic carbon sequestration, dark carbon fixation (DCF) mediated mainly by chemoautotrophic microorganisms has recently been recognized as an important carbon sink process in coastal wetlands [3–6]. Chemoautotrophic microbes are known to synthesize organic molecules from dissolved inorganic carbon via seven different pathways, including Calvin–Benson–Bassham (CBB) cycle, reductive acetyl-CoA (W-L) pathway, 3-hydroxypropionate/4-hydroxybutyrate (3HP/4HB) cycle, reductive tricarboxylic acid (rTCA) cycle, 3-hydroxypropanoate (3HP) bicycle, dicarboxylate/4-hydroxybutyrate (DC/4HB) cycle, and reductive glycine pathway [7–9]. Utilizing these carbon sequestration pathways, chemoautotrophs in estuarine and coastal ecosystems were estimated to assimilate ~328 Tg C yr^−1^, representing ~42.6% of oceanic DCF production [10].

Under the context of global climate change, coastal wetlands and their capacity for carbon sequestration has been the frontline of global warming due to the influence of various complex factors, such as land–sea interaction and intensive anthropogenic disturbance [6, 11, 12]. Many field experiments have been established in recent decades to investigate the response of ecosystems to climate warming [13–15] and the sensitivity of vulnerable coastal ecosystems to climate change has been stressed [16–18]. A warming climate was suggested to induce environmental changes that accelerate the microbial degradation of organic carbon and the release of carbon dioxide (CO_2_) [19]. However, water loss induced by temperature rising may offset the warming effect, slowing down the rate of soil CO_2_ release to pre-warming levels [20, 21]. A study of *in situ* warming spanning over 50 yr showed that both organic carbon uptake and respiration rates tended to be lower at 1.5°C warming levels than in ambient field plots [14]. Nevertheless, a meta-analysis of carbon-cycling responses to global change suggested that experimental warming slightly increased belowground net primary productivity [15]. Furthermore, limited field studies showed that DCF rates were positively [22, 23] or negatively [24, 25] correlated with temperature increase. These results suggest an urgent need to better understand the complex feedback mechanisms of soil carbon retention and loss under warming stress [14]. Moreover, the response of dark carbon sequestration to global warming and its microbial coping strategies remains largely unclear [15, 26, 27], which greatly hinders an accurate quantitative assessment of coastal net carbon sequestration capacity in the context of climate change.

We predicted that warming may greatly affect the activity of chemoautotrophic microbes and thus alter the carbon sequestration through DCF in coastal wetlands, as temperature is a primary factor regulating microbial metabolic activities. Furthermore, it was hypothesized that different chemoautotrophic microbial populations would exhibit differential responses to climate warming, potentially leading to alterations in chemoautotrophic microbial communities and their carbon fixation pathways. To test these hypotheses, we utilized metagenomics and carbon isotope tracing technologies to determine the feedback mechanisms of chemoautotrophs in response to climate warming and their role in governing carbon sink in coastal wetlands based on a 4-yr field warming experiment. This study is important for improving the prediction of carbon sink capacity of the coastal blue carbon ecosystem under a warming climate.

## Materials and methods

### Study area and plot set-up

The field warming experiments were conducted at the Yangtze Estuary Wetland Ecosystem Field Station (YEWEFS), which is situated on the eastern boundary of the Chongming Island, China (31°38' N, 121°58′ E) (Fig. S1). The study area experiences a northern subtropical monsoon climate, with an average annual temperature of 15.3°C and precipitation of 1004 mm [28]. We established an open-top chambers (OTCs) warming system to simulate the effects of global climate warming on the carbon cycle in a salt wetland dominated by native wetland vegetation, *Phragmites australis* (*P. australis*) [29]. The experiment included ambient control and warming treatments with an average annual temperature increase of ~1.5°C. Four plots were set for each treatment. These OTCs, 3.5 m high and 12.5 m^2^ in area, were made of fluorinated glass to raise temperature (Fig. S1). The air temperature was recorded using a HOBO TidbiT v2 Temperature Data Logger (Onset, USA), which was placed 1.2 m above the ground. Additionally, the soil temperature at a depth of 5 cm below the ground surface was also monitored.

### Soil sampling

After 4 yr of field warming, a total of 64 soil samples were collected from both the control and warming plots during four seasons: summer (July 2022), autumn (November 2022), winter (January 2023), and spring (April 2023). Soils samples were obtained from the surface layer [0–5 cm, A horizon (Ah layer)] and the subsurface layer [5–10 cm, B horizon (Bh layer)] of each plot, using a 5-cm-diameter screw drill and sterile polyethylene ziplock bags. Immediately after collection, all soil samples were placed into an insulated box with ice packs and transported to the laboratory within 3 hours. Upon arrival at the laboratory, soil samples from each plot were thoroughly mixed in an anoxic chamber filled with nitrogen to create a composite sample. Approximately 10 g of each soil sample was collected using sterile cryopreservation tubes, flash-frozen in liquid nitrogen, and stored at −80°C for subsequent microbial analysis. The remaining portion of each sample (~1000 g) was stored at 4°C for the determination of DCF rates and soil biogeochemical properties.

### Measurements of soil physicochemical properties

Soil pH and electrical conductivity (EC) were determined at a soil-to-water ratio of 2.5:1, using a pH meter equipped with a calibrated combined glass electrode (Mettler-Toledo, Switzerland) and an EC meter (Shanghai REX, China), respectively [30]. Soil water content was estimated based on weight loss of soil after freeze-dried. Soil grain size was measured using a LS 13 320 laser diffraction particle size analyzer (Beckman-Coulter, USA). Total organic carbon (TOC) and total nitrogen (TN) of soil were determined using an elemental analyzer (Vario Macro CNS, Germany) after acidification with 1 M HCl [31]. Exchangeable inorganic nitrogen [ammonium (NH_4_^+^), nitrite (NO_2_^−^), and nitrate (NO_3_^−^)] was extracted from the soil using 2 M KCl and subsequently determined using a continuous-flow nutrient analyzer (SAN plus, Skalar Analytical B.V., the Netherlands) [30].

### Measurement of dark carbon fixation rates

DCF rates of soil were determined using the ^13^C-CO_2_ labeling method [32–34]. Briefly, soil sample (20 g) was placed into a 120 ml serum bottle. The bottles were sealed with butyl rubber septa and aluminum caps and pre-incubated in the dark at near *in situ* temperature for 48 h. Following the pre-incubation period, all bottles were opened, and the headspace was flushed with synthetic air (75% N_2_ and 25% O_2_ for Ah layer; 100% N_2_ for Bh layer) for 5 min. Subsequently, the bottles were sealed immediately. 5% (v/v) ^13^CO_2_ was added using syringes to the experimental bottles, and an equal volume of ^12^CO_2_ was added to the control groups. The samples were incubated in the dark at near *in situ* temperature for 8 days (Fig. S2). During the incubation, the headspace of the bottles was flushed with synthetic air every 2 days, and the ^13^CO_2_ or ^12^CO_2_ were re-injected. After incubation, the samples were freeze-dried and the DCF rates were determined by TOC and δ^13^C values of the samples (Supplementary method).

### DNA extraction and quantitative PCR

Total genomic DNA of the soil samples was extracted utilizing the DNeasy PowerSoil Kit (QIAGEN, Germany). Subsequently, DNA concentrations were determined using a NanoDrop-2000 spectrophotometer (Thermo Scientific, USA). The genes encoding the large subunit of the RuBisCO Form I and Form II enzymes (*cbbL* and *cbbM*, CBB cycle), the alpha subunit of acetyl-CoA carboxylase (*accA*, 3HP/4HB cycle), the 4- hydroxybutyryl- CoA dehydratase (*hbd*, 3HP/4HB cycle and DC/4HB cycle), and the beta-subunit of the ATP-dependent citrate lyase (*aclB*, rTCA cycle) were quantified with the primers K2f/V2r [35], cbbM-f/cbbM-r [36], Crena_529F/Crena_981R [37], qPCR_hcd_f/qPCR_hcd_r [38], and aclB_402F/aclB_907R [6], respectively (Table S1). Additionally, the bacterial and archaeal abundances were determined by quantifying the 16S rRNA gene using the primers 341F/518R [39] and Arch-967F/Arch-1060R [40], respectively. The quantitative PCR (qPCR) procedures were executed on the ABI7500 Sequence Detection System (Applied Biosystems, Canada) using the SYBR green method, and the qPCR thermal cycling conditions are detailed in Table S1. Negative controls without DNA were also performed. In this study, qPCR results meeting the criteria of a single melting-curve peak, amplification efficiencies exceeding 90%, and correlation coefficients surpassing 0.98 were employed for calculating gene abundance. Gene abundances were normalized based on soil dry weight and copies of 16S rRNA genes, respectively.

### PCR amplicon sequencing and data analysis

The modified V4 16S rRNA gene primers 515F (5'-GTGYCAGCMGCCGCGGTAA-3′) and 806R (5'-GGACTACNNGGGTATCTAAT-3′) [41] were used to simultaneously analyze the bacterial and archaeal communities in the same dataset. Triplicated PCR amplicons were purified (Agencourt AMPure XP, Beckman Coulter, Brea, CA), quantified (Qubit2.0 DNA, Life Technologies), and mixed with an equimolar concentration. The products were then sequenced on a MiSeq system (Illumina, USA). The raw sequencing reads were demultiplexed based on their unique barcodes using QIIME2 [42]. The paired-end reads for each sample were merged, and an average of 65 886 ± 17 309 sequences were then denoised using DADA2 [43] to obtain amplicon sequence variants (ASVs). The ASVs were classified into taxonomic lineages using the Ribosomal Database Project classifier [44] against the SILVA v138 database at a minimum confidence level of 0.7. Each sample was resampled to the same depth of 31 340 sequences in subsequent comparative analyses. The representative 16S rRNA gene sequences were aligned using MAFFT(L-INS-I) v.7.429 [45] and pruned using trimAI v.1.4 [46]. Maximum-likelihood phylogenetic tree of 16S rRNA gene sequences were constructed using IQ-TREE v.2.2.2.6 [47] with GTR + F + I + G4 model.

### Metagenome sequencing and analysis

Metagenomic sequencing was perform on all samples to analyze soil microbial functional composition. Genome libraries were constructed using the NEXTFLEX Rapid DNA-Seq kit and paired-end sequenced using NovaSeq Reagent Kits on the NovaSeq platform (2 × 150 bp) (Illumina, CA, USA) provided by Majorbio (Shanghai, China). The resulting metagenomic library sizes ranged from 10.7 to 23.6 Gbp (mean = 16.9 Gbp; Table S2). Low-quality reads and adaptors were removed by trimming raw reads using fastp v0.20.0 (length < 50, quality <20) [48]. Subsequently, the trimmed sequences were individually de novo assembled with MEGAHIT v.1.2.9 (--k-min 27, --k-max 127, --k-step 10) [49]. Open reading frames (ORFs) were predicted from the acquired contigs using Prodigal v.2.6.3 [50], and a nonredundant gene catalog was constructed using CD-HIT v.4.8.1 (-aS 0.9, −c 0.9) [51]. The identified genes were then annotated with biological functions using DIAMOND v.2.0.13 [52] (e-value <1e-5, bit score > 50) by comparing against the KEGG database [53]. Functional genes associated with different carbon fixation pathways (Table S3) [54] and organic carbon degradation (Table S4) [55, 56] were extracted. High-quality reads were mapped back to ORFs using Bowtie2 v.2.3.5.1 [57] to determine the coverage information for these functional genes in each sample. The target reads were filtered using CoverM v0.6.1 (https://github.com/wwood/CoverM) (identity >90%, aligned length > 50%), and the resulting reads were aligned to NCBI archaeal and bacterial RefSeq database using Kraken2 v.2.1.3 [58]. The composition of chemoautotrophic communities was determined by key marker enzymes of various carbon fixation pathways (Table S3). The normalized abundances of functional genes and microbes across different metagenomes were quantified using counts per million (CPM), which represents the number of target reads per million reads.

### Statistical analysis

The normality of variables, including soil geochemical properties, DCF rates, and functional gene abundances, was checked using Shapiro–Wilk test, and the homogeneity of variances for these variables was checked using two-tailed F test. To assess possible variations, either Student's t-test or Mann–Whitney test was performed using the *rstatix* R package [59]. The relationship between DCF rates and temperature was analyzed using the square root model, Q10 and T_min_ [60], which were used to assess the temperature adaptation of chemoautotrophic communities driving the DCF process. T_min_ in different treatments was calculated using the equation: ${R}_{DCF}={\left[a\times \left(T-{T}_{min}\right)\right]}^2$, where *R_DCF_* is the measured DCF rate at the temperature T °C, *T_min_* is the calculated minimum temperature for the activity, and *a* is a slope parameter without any direct biological meaning. Variations in Q10 in different treatments were calculated using the equation: $Q10=\left[\Big(T+10-{T}_{min}\right)/\left(T-{T}_{min}\right)\Big]{}^2$. Nonlinear regression with generalized additive model (GAM) was conducted to investigate the relationship between DCF rates and environmental variables including temperature and water content (Table S5), using the *mgcv* R package [61]. The richness (Chao1 index), diversity (Shannon index), and evenness (Pielou index) of microbial community were calculated using the *vegan* R package [62]. In addition, principal coordinates analysis (PCoA) and permutational multivariate analysis of variance (PERMANOVA) were performed to assess the impact of climate warming and changes in environmental parameters on the microbial communities.

Considering the plot design, multi-season sampling, and repeated measurements, linear mixed-effects models (LMMs) were utilized through the *lme4* R package [63] to assess the effects of climate warming on DCF rates, diversity of prokaryotic microbial communities based on 16S rRNA gene sequencing, and the normalized abundances of chemoautotrophic communities using different carbon fixation based on metagenomic sequencing. These variables were standardized using 'scale' function in R (version 4.3.1). In the LMMs, warming was treated as a fixed effect, and sampling season, layer, and plot were considered as random intercept effects. The regression coefficients (β) and 95% confidence interval (CI) in the LMMs were used to represent the impact of climate warming. The random effects variance in LMMs due to different seasons was calculated using 'VarCorr' function in the *lme4* R package. Wald type II χ^2^ tests, conducted using the *car* R package, were used to calculate the *P* values in the LMMs [64]. LMMs and structural equation modeling (SEM) were further employed to investigate the influence mechanisms of climate warming and subsequent environmental changes on DCF process (Supplementary method). The statistical significance level for all analyses was set at *P* < 0.05.

## Results

### Effects of warming on soil geochemical factors and microbial communities

**Figure 1:**
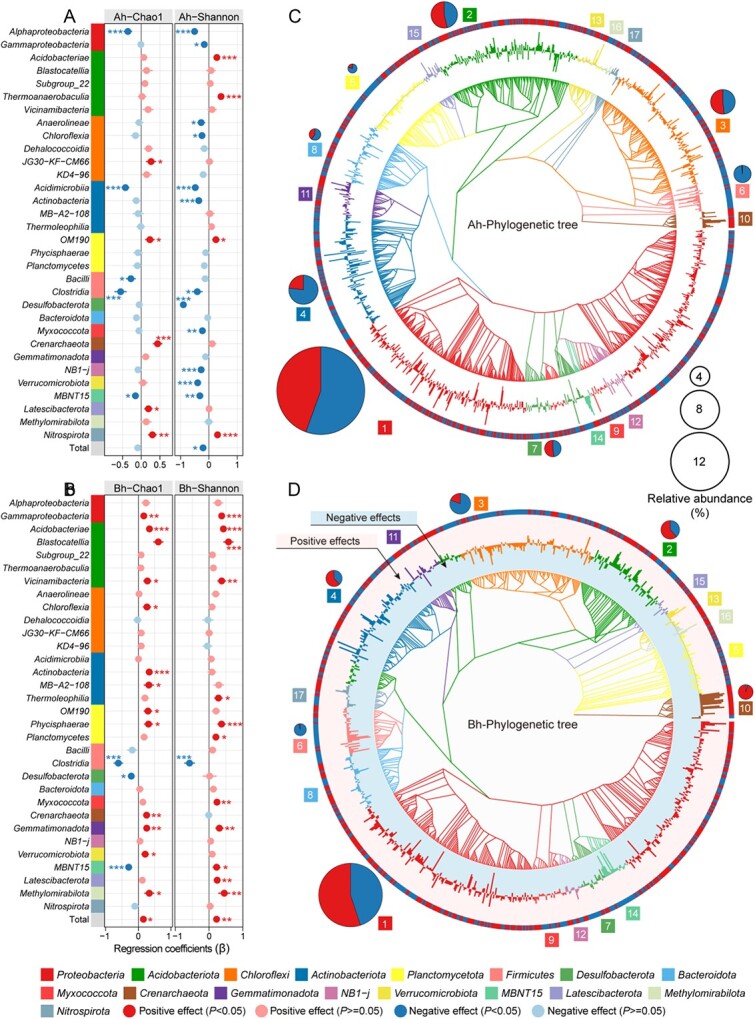
Effects of warming on various microbial lineages in coastal wetland soils. (a–b) The regression coefficients (β) of warming on the richness and diversity of major microbial lineages, determined through LMMs based on 16S rRNA gene sequences. Statistical significance was determined using Wald type II χ^2^ tests (*n* = 96). The results are presented as the mean ± standard deviation (SD) of the coefficients. Significance levels are indicated by asterisks (^*^P < 0.05, ^*^^*^P < 0.01, and ^*^^*^^*^P < 0.001). Nonsignificant changes are denoted by light-colored dots. Ah and Bh represent surface (0–5 cm) and subsurface (5–10 cm) layers of the soil, respectively. (c–d) Maximum-likelihood tree of individual microbial 16S rRNA gene ASVs (the innermost ring), where only ASVs with a significant (*P* < 0.05) response to warming and an average reads number > 2 among samples are included. The outside and inside bars of the second ring represent the positive and negative effect sizes of warming on rescaled taxon relative abundances, respectively. The colors of the branches in the first ring and the bars in the second ring correspond to individual phyla. Additionally, colors in the third ring represent ASVs with significant increase or decrease under warming. The size of the pies reflects the overall relative abundance (>2%) of microbial phyla across all samples, with different colored sections representing the proportions of the total abundance of ASVs that increased or decreased under warming, respectively.

During the 4-yr field warming experiment, the average annual air temperature in the OTCs increased by ~1.5°C, and the average annual soil temperature increased by ~0.6°C (Fig. S3). LMMs results showed that warming had significant positive effects on soil pH (β = 0.69 and 0.90, *P* < 0.05) and NO_3_^−^ content (β = 0.34 and 0.78, *P* < 0.05; Fig. S4a and Table S6). Soil water content in the warming treatment exhibited varying degrees of decrease compared with the control plots (Fig. S4c). Soil EC in the warming treatment decreased by 6.1 to 10.6% (Fig. S4d). Soil TOC content increased significantly in the warming treatment compared with the ambient control during autumn (*P* < 0.05; Table S7). However, in other seasons, TOC content showed a decreasing trend (Fig. S4i). Other measured properties, including NH_4_^+^, NO_2_^−^, and TN, did not exhibit a clear response pattern under warming condition (Fig. S4). PCoA results based on 16S rRNA gene sequences showed that warming significantly altered the microbial community structure in both Ah and Bh layer soils (Fig. S5). Additionally, the PERMANOVA analyses showed that soil parameters, including water content, EC, pH, NO_2_^−^, and TOC content, had significant effects on the composition of the microbial community (Table S8). LMMs analysis revealed that warming had different effects on microbial biodiversity at different soil depths (Fig. 1; Table S9). The warming treatment resulted in a decrease in microbial richness (β = −0.09) and diversity (β = −0.21, *P* < 0.05) in the Ah layer. In contrast, it led to a significant increase in the richness (β = 0.16, *P* < 0.01) and diversity (β = 0.22, *P* < 0.01) of microbes in the Bh layer (Fig. 1a–b). These effects of climate warming on microorganisms at different soil layers of coastal wetlands were also evident at individual microbial ASV level. Under experimental warming, the relative abundance of *Proteobacteria* and *Actinobacteriota* lineages decreased by 55.5 and 76.8% in the Ah layer, respectively. Conversely, in the Bh layer, the relative abundance of *Proteobacteria* and *Actinobacteriota* lineages increased by 55.0 and 62.1%, respectively (Fig. 1c–d). However, nearly all ASVs of *Crenarchaeota* (99.5 and 95.7% in the Ah and Bh layers, respectively) exhibited an increasing trend under experimental warming, whereas almost all ASVs of *Firmicutes* (98.3 and 96.0% in the Ah and Bh layers, respectively) were negatively affected by warming (Fig. 1c–d).

### Effects of warming on DCF rates

**Figure 2:**
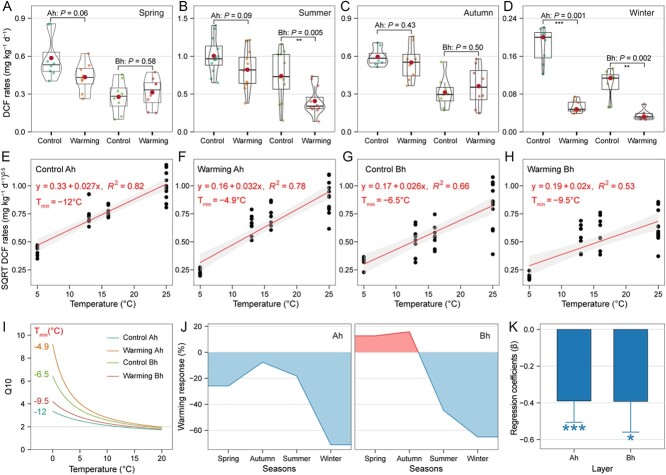
DCF rates of coastal wetland soils in response to climate warming. (a–d) Effects of climate warming on DCF rates. Boxes represent the interquartile range (IQR) between the first and third quartiles (25th and 75th percentiles, respectively), and the horizontal line inside the box defines the median. Whiskers represent the lowest and highest values within 1.5 times the IQR from the first and third quartiles, respectively. Ah and Bh represent surface (0–5 cm) and subsurface (5–10 cm) layers of the soil, respectively. (e–h) Square root (SQRT) relationship between temperature and DCF rates in both Ah and Bh layers of warming and control plots. (i) Variation in Q10 with temperature calculated for different T_min_ from fitted curves of plots e-h. (j) Responses of DCF rates to climate warming. (k) The regression coefficients (β) of warming on the DCF rates in Ah and Bh layers, determined through LMMs. Significance levels are denoted by asterisks (^*^P < 0.05, ^*^^*^P < 0.01, and ^*^^*^^*^P < 0.001).

Generally, DCF rates in coastal wetlands were greatly reduced by climate warming (Fig. 2; Table S10). In the Ah layer, DCF rates decreased by 7.6 to 71.2% throughout the year (Fig. 2). However, in the Bh layer, DCF rates slightly increased in spring and autumn (12.7 and 15.8%, respectively, *P* > 0.05), but significantly decreased in summer and winter (44.7 and 64.1%, respectively, *P* < 0.05; Fig. 2). LMMs analysis showed that climate warming had significant negative effects on DCF rates in both Ah (β = −0.39, *P* < 0.001) and Bh (β = −0.39, *P* < 0.05) layers. In the ambient control, DCF rates in summer (0.15–1.41 mg kg^−1^ d^−1^) were 5–7 times higher than those in winter (0.05–0.22 mg kg^−1^ d^−1^), whereas in the warming plots, DCF rates in summer (0.14–1.21 mg kg^−1^ d^−1^) were 11.5–15.5 times higher than those in winter (0.03–0.07 mg kg^−1^ d^−1^). Q10 and T_min_ were further used to assess the differences in temperature adaptation of chemoautotrophic communities between the ambient control and warming plots. In the Ah layer, DCF process had a higher T_min_ in the warming plots (T_min_ = −4.9°C, *R*^2^ = 0.78; Fig. 2f) than that in the control plots (T_min_ = −12°C, *R*^2^ = 0.82; Fig. 2e), which led to a higher Q10 in the warming treatments (Fig. 2i). In contrast, in the Bh layer, DCF activity showed a lower T_min_ in the warming plots (T_min_ = −9.5°C, *R*^2^ = 0.82; Fig. 2h) than that in ambient control (T_min_ = −6.5°C, *R*^2^ = 0.82; Fig. 2g), resulting in a lower Q10 under warming condition in this deep soil layer (Fig. 2i).

### Effects of warming on chemoautotrophs and carbon fixation pathways

**Figure 3:**
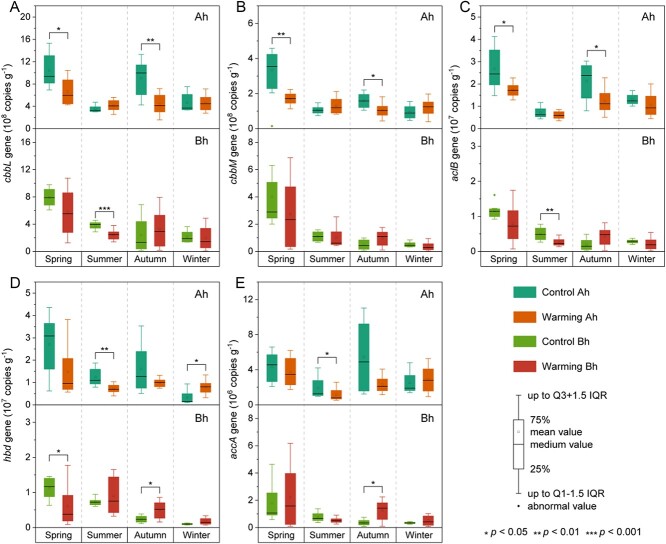
Abundances of *cbbL* (a), *cbbM* (b), *aclB* (c), *hbd* (d), and *accA* (e) genes in coastal wetland soils. The abundances were normalized based on soil weight. Ah and Bh represent surface (0–5 cm) and subsurface (5–10 cm) layers of the soil, respectively. Boxes represent the IQR between the first and third quartiles (25th and 75th percentiles, respectively), and the horizontal line inside the box defines the median. Whiskers represent the lowest and highest values within 1.5 times the IQR from the first and third quartiles, respectively. The asterisk above the column denotes significant differences between control and warming treatments (^*^P < 0.05, ^*^^*^P < 0.01, and ^*^^*^^*^P < 0.001).

**Figure 4:**
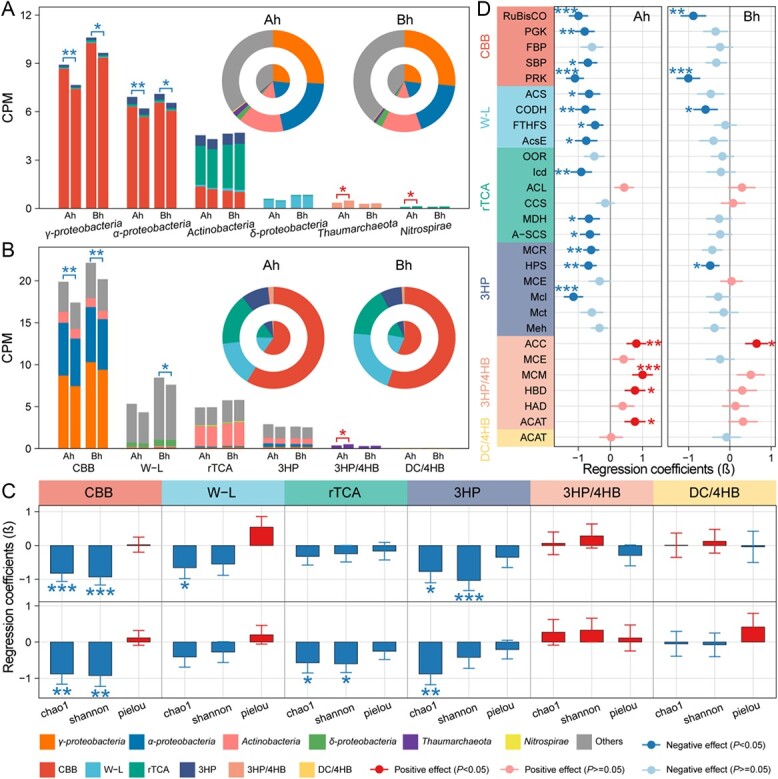
Effects of warming on chemoautotrophic community and their carbon fixation pathways in coastal wetland soils. (a–b) The normalized abundances (CPM) of various chemoautotrophic microbial lineages and carbon fixation pathways based on the metagenome analysis. Functional genes were annotated by comparing against the KEGG database. Ah and Bh represent surface (0–5 cm) and subsurface (5–10 cm) layers of the soil, respectively. The left and right bars represent the control and warming groups, respectively. The asterisks above the bar denote significant effect of warming on the normalized abundance of chemoautotrophs based on LMMs. The inside and outside rings in the panel represent the proportions of the abundance for various chemoautotrophic microbial lineages or carbon fixation pathways in the control and warming groups, respectively. (c) The regression coefficients (β) of climate warming on the richness (Chao1), diversity (Shannon), and evenness (Pielou) of chemoautotrophic communities using different carbon fixation pathways. (d) Effects of warming on the normalized abundance of enzymes associated with different carbon fixation pathways were determined using LMMs. Significance levels are denoted by asterisks (^*^P < 0.05, ^*^^*^P < 0.01, and ^*^^*^^*^P < 0.001).

The qPCR results showed that the abundance of *cbbL* and *cbbM* genes involved in the CBB cycle primarily exhibited a negative response to climate warming, with reductions ranging from 1.3 to 51.7% (Fig. 3a–b; Table S11). *AclB* gene abundance associated with the rTCA cycle decreased by 15.4 to 47.7% in the warming plots, with the exception of Bh layer in autumn (Fig. 3c). The abundance of *hbd* and *accA* genes involved in the 3HP/4HB cycle decreased by 13.1 to 57.5% in the Ah layer (excluding winter), but tended to be increased in the Bh layer in response to climate warming (Fig. 3d–e). These response patterns of functional genes to climate warming based on gene abundances normalized by soil weight were generally consistently with the normalization according to 16S rRNA gene copy numbers (Fig. S6). Metagenomics revealed that chemoautotrophs in coastal wetlands were primarily affiliated with *Proteobacteria*, specifically γ-*proteobacteria* (26.5%, given as the average across all sampling sites), α-*proteobacteria* (19.4%), and δ-*proteobacteria* (1.9%) (Fig. 4a and Fig. S7). The LMMs analysis showed that simulated warming had significant negative effects on γ- and α-*proteobacteria* in both the Ah and Bh layers of soils (β = −0.91 to −0.59, *P* < 0.05) (Fig. 4a; Table S12)*.* However, it had significant positive effects on *Thaumarchaeota* and *Nitrospirae* in the Ah layer (β = 0.61–0.77, *P* < 0.05).

Under simulated warming, the CPM of chemoautotrophs with the CBB cycle, W-L pathway, and 3HP bicycle decreased by 3.7 to 19.1%, whereas that of 3HP/4HB cycle increased by 11.7 to 39.3% (Fig. 4b; Table S13). Consistently, climate warming showed significant negative effects on the biodiversity of chemoautotrophic communities of the CBB cycle, W-L pathway, and 3HP bicycle, but had significant positive effects on that of the 3HP/4HB cycle (Fig. 4c; Table S14). Although warming slightly increase the CPM of chemoautotrophs using the rTCA cycle, their biodiversity was decreased (Fig. 4b–c). Furthermore, the CPM of enzymes associated with the CBB cycle, W-L pathway, and 3HP bicycle was significantly decreased in the warming plots compared with the ambient control (Fig. 4d and Fig. S8; Table S15). In contrast, the CPM of enzymes associated with the 3HP/4HB cycle showed a positive response to climate warming. However, the CPM of enzymes associated with the rTCA and DC/4HB cycles did not exhibit a significant response to climate warming. These results suggested that warming had differential impacts on different carbon fixation pathways in coastal wetlands (Fig. 4d and Fig. S8).

### Controlling mechanisms of DCF under climate warming

**Figure 5:**
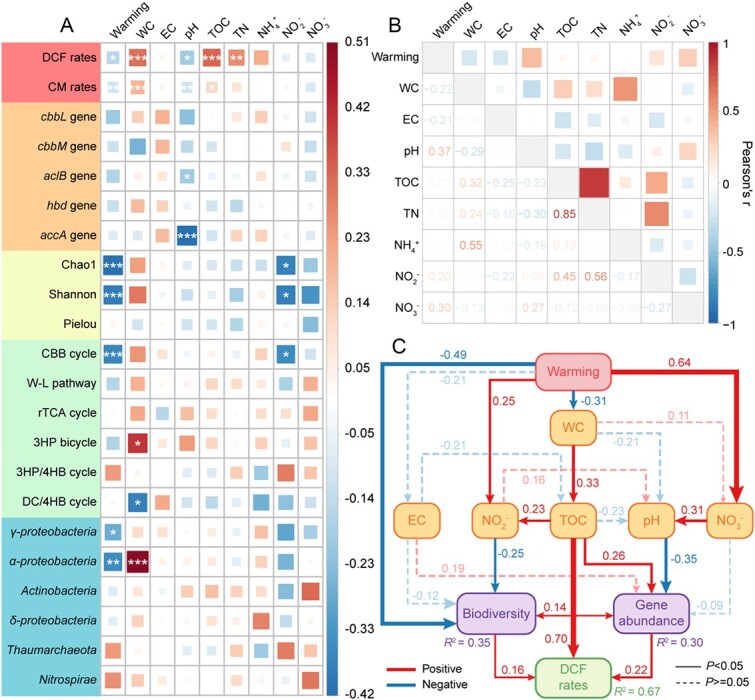
Environmental factors influencing variations in the DCF rates process and associated chemoautotrophs under climate warming. (a) Correlations between environmental parameters and DCF rates, CM rates, abundances and diversity of chemoautotrophic microbial communities, carbon fixation pathways, and majority lineages of chemoautotrophs, determined using LMMs. The color denotes the correlation coefficient statistical significance was assessed using Wald type II χ^2^ tests with *n* = 64 independent soil samples. Significance levels are denoted by asterisks (^*^P < 0.05, ^*^^*^P < 0.01, and ^*^^*^^*^P < 0.001). (b) Pairwise correlations between environmental parameters in coastal wetland soils. The numbers in the lower triangle represent the Pearson’s correlation coefficient. The shade of color and the size of square represent strength of the correlation. (c) SEMs assessing the relationship between environmental variables, abundance and diversity of chemoautotrophic microbial communities, and DCF rates in coastal wetland soils. Model parameters: df = 22, χ^2^ = 20.3 *P* = 0.57, gfi = 0.95, cfi = 1, rmsea = 0, srmr = 0.05. Solid lines represent significant relationships, and dashed lines represent nonsignificant relationships. Numbers near the arrow indicate the standardized effect coefficients. *R*^2^ represents the proportion of variance explained for dependent variable.

DCF rates were positively correlated with soil water content, TOC and TN contents (LMMs *r* ranging from 0.26 to 0.33, *P* < 0.05), but negatively correlated with soil pH (LMMs *r* = −0.19, *P* < 0.05) (Fig. 5a; Table S16). Negative correlations were also observed between soil pH and the abundance of chemoautotrophic functional genes (LMMs *r* ranging from −0.42 to −0.05). However, the diversity and richness of chemoautotrophs were positively related to soil water content (LMMs *r* ranging from 0.24 to 0.30), but showed significantly negative correlation with climate warming (LMMs *r* ranging from −0.41 to −0.39, *P* < 0.05) (Fig. 5a; Table S16). SEM analysis was further performed to explore how climate warming affects DCF process by changing environmental variables and chemoautotrophic microbial communities (Fig. 5c; Table S17). Climate warming was identified as a primary factor indirectly decreasing DCF rates, with a standardized effect coefficient (*b*) of −0.17 (*P* < 0.01; Fig. S9). The most significant factors directly affecting DCF rates were recognized as TOC (*b* = 0.70, *P* < 0.001), chemoautotrophic microbial abundance (*b* = 0.22, positively related to soil TOC, water content, negatively affected by climate warming, pH), and biodiversity (*b* = 0.16, negatively correlated with climate warming and NO_2_^−^ content) of chemoautotrophs (Fig. 5c; Table S17).

## Discussion

Understanding the response of coastal carbon cycling to climate warming is of great significance for predicting future climate change [65]. The feedback of ecosystem carbon cycling to climate warming consists of two scenarios, one is the release of additional CO_2_, which accelerates climate warming [19, 66], whereas the other is the increase of carbon fixation thus alleviating global warming [15, 67]. DCF process, conducted mainly by chemoautotrophs, contributes greatly to primary production and the global carbon budget [33]. Exploring the effects of climate warming on DCF process in coastal wetlands is of great practical importance for model optimization and climate change prediction.

Chemoautotrophs are known to conduct DCF process by oxidizing reductive substrates [e.g. hydrogen (H_2_), ammonia (NH_3_), NO_2_^−^, hydrogen sulfide (H_2_S), sulfur (S), thiosulfate (S_2_O_3_^2−^), and ferrous iron (Fe^2+^)], initiating a chain of electron-transfer reactions that culminate in the reduction of terminal electron acceptors (TEAs, such as O_2_, NO_3_^−^). Using metabolic energy obtained from these redox reactions, they assimilate inorganic carbon and fix it into organic molecules [68]. Environmental changes commonly impact the DCF process by influencing the availability of reductive substrates and TEAs, as well as the abundance and biodiversity of chemoautotrophs [4, 5]. Temperature has been recognized as a primary factor regulating microbial metabolic activities [6, 12, 14].

Following 4 yr of field simulated warming (an average annual air temperature increase of 1.5°C), the potential DCF rates declined significantly (Fig. 2). However, DCF rates increased with temperature during different seasons of the year, both in the ambient control and the warming treatment plots (Fig. 2e–h). The seasonal variation of DCF rates can be attributed to the increase of chemoautotrophic activity within the optimum temperature (T_opt_) in each plot [6, 23]. Nevertheless, the decrease of DCF activities under climate warming compared with the ambient control might be attributed to the alterations in abiotic environmental conditions and biotic interactions resulting from climate warming. We estimated that the reduction of DCF carbon sequestration in global coastal marshes ranged from 0.08 to 1.5 Tg C yr^−1^ (area: 0.02–0.40 × 10^12^ m^2^; soil depth: 10 cm), equivalent to 1.7% of the annual carbon buried in salt marsh ecosystems [69].

Climate warming led to an obvious reduction in soil water content throughout the year (Fig. S4c), which may contribute to the decrease of DCF rates in the simulated warming plots. GAM analysis showed that DCF activities exhibited a pyramid-shaped response surface with temperature and water content (Fig. S10), and a lower fitted coefficient was observed in the simulated warming condition (Table S5). This result indicated that the increasing rate of DCF activities with the increase of seasonal soil temperature was generally reduced by the decrease of water content under climate warming (Figs S4 and S10). Previous studies have also demonstrated that soil moisture played an important role in regulating the variability in net primary productivity and shaping microbial richness and diversity [67, 70]. Quan et al. [71] demonstrated that warming exhibited a stimulating effect on net carbon uptake (negative feedback to climate warming) in wet conditions, but showed a depressing effect (positive feedback to climate warming) in dry conditions. The decrease of water content in coastal wetland soils under climate warming may lead to a reduction in the mineralization of organic matter (Fig. 5c and Fig. S11) [72, 73], thus reducing the availability of reductive substrates for the activity of chemoautotrophs.

The relatively low carbon mineralization (CM) rates (Supplementary method) and the resulting decrease in the availability of reductive substrates for DCF in the warming plots may also be related to the generally reduced soil TOC contents under climate warming (Fig. 5a and Fig. S4i). Melillo et al. [66] also reported that long-term warming resulted in a decrease of organic matter and less organic carbon available to microorganisms. In this study, the TOC content in the warming plots was significantly higher compared with the ambient control in autumn, which could be due to the release of more organic carbon into the soil from the roots of *P. australis* under climate warming, as relatively high below-ground biomass of *P. australis* (Supplementary method) was detected in the warming plots during this season (Fig. S12). Nevertheless, despite the higher TOC contents, CM activities were also reduced under climate warming in autumn (Figs S4i and S11). Previous studies suggested that warming may increase the proportion of soil recalcitrant carbon [13], which may lead to lower CM rates during the fall season in the warming plots. However, the CM rates in the present study were determined in the absence of live roots and may therefore be underestimated as the *in situ* root exudates could accelerate the decomposition of soil organic carbon via rhizosphere priming effect [74]. This hypothesis was supported by the metagenomic data, which showed that most of the genes associated with heterotrophic soil respiration slightly increased in autumn under climate warming (Fig. S13) [13]. This assumption could also explain the significant decline in TOC contents from autumn to winter in the warming plots. However, further studies on the structure and *in situ* decomposition rate of soil organic carbon are still required. Overall, this study demonstrated that the soil TOC contents in coastal wetlands were generally reduced on an annual scale under climate warming, and a lower carbon sequestration rates through DCF would, in turn, reduce chemoautotrophic microbial source organic carbon in the warming plots. Therefore, considering that there was no significant change in total photosynthetic biomass of *P. australis* between the warming and control plots (Fig. S12), we roughly inferred that warming might lead to a decrease in carbon storage in coastal wetlands.

The LMMs results showed that climate warming had a negative effect on DCF rates in both the Ah and Bh layers (Fig. 2k). However, the negative effect was weaker in the Bh layer (an annual decrease of 20.3%) compared with the Ah layer (an annual decrease of 30.7%) (Fig. 2j). This result can be intuitively interpreted by T_min_ and Q10, which were indicators for temperature sensitivity of chemoautotrophs [60]. In the Ah layer, climate warming increased T_min_ and thus Q10-value (Fig. 2i), indicating that chemoautotrophs increased their intrinsic temperature sensitivity under the long-term climate warming [60], probably because of the changes in microbial community composition [13]. Conversely, in the Bh layer, T_min_ and Q10-value in the warming plots were lower compared with the ambient control, implying the enhancement of thermal adaptation by simulated warming [75, 76]. In fact, DCF rates in the Bh layer were slightly higher, though not significantly, in the warming plots than those in the ambient control during spring and autumn (Fig. 2a, c). From the perspective of microbial diversity, under warming conditions, the microbial biodiversity in the Ah layer significantly decreased, which was consistent with previous finding in grassland soil as previously described [70], whereas the microbial biodiversity in the Bh layer significantly increased (Fig. 1). Nevertheless, on an annual scale, carbon sequestration via DCF in the Bh layer under simulated warming was reduced, which might be attributed to the reduction in TOC content and the resulting lower availability of reductive substrate for DCF activities (Fig. S14). However, the inconsistent feedback mechanism of chemoautotrophic microbes to climate warming at different soil depths to warming requires further study.

Compared with abiotic environmental conditions, the most direct causative factor for the decline of DCF rates in coastal wetlands under climate warming was recognized to be the decrease of the abundance and biodiversity of chemoautotrophic microbial community (Fig. 5). Biodiversity of chemoautotrophs utilizing CBB cycle, W-L pathway, rTCA cycle, and 3HP bicycle decreased significantly under climate warming (Fig. 4c). In addition, the relative abundance of chemoautotrophs such as γ-, α-, and δ-*Proteobacteria* also decreased to varying degrees in the warming plots (Fig. 4a). These results may therefore contribute to the decrease of DCF rates under warming stress. Consistently, previous studies have also reported that the reduction of microbial diversity due to climate warming led to the decline of ecosystem functions such as gross ecosystem productivity, ecosystem respiration, and net carbon sink [70, 77, 78]. However, climate warming exhibited a significant positive effect on the biodiversity of *Thaumarchaeota* utilizing the 3HP/4HB cycle (i.e. ammonia-oxidizing archaea; Fig. 4a), which coincided with previous results of warming experiments [79, 80]. Moreover, the relative abundance of enzymes associated with the 3HP/4HB cycle showed a positive response to climate warming (Fig. 4d and Fig. S8). These results suggest that the relative contribution of the 3HP/4HB cycle to DCF will increase in coastal wetlands in a warming world. In contrast, the relative importance of the dominant DCF pathways (CBB cycle and W-L pathway) may be reduced due to warming stress (Fig. 4), thus altering the pattern of DCF carbon sequestration pathways in coastal wetlands.

In summary, based on a 4-yr field warming experiment, the feedback mechanisms of chemoautotrophs to climate warming and the carbon sink they govern in coastal wetlands were revealed utilizing metagenomics and carbon isotope tracing technologies. Results showed that climate warming reduced the chemoautotrophic carbon sink of coastal wetlands, thus aggravating the reduction of soil carbon storage and causing a positive feedback to climate warming. It was estimated that global coastal marshes will lose up to 0.08–1.5 Tg C yr^−1^ due to a decrease in DCF rates under a temperature increase of ~1.5°C. Results also revealed that the intrinsic microbial temperature sensitivity and environmental changes jointly determined the warming effect on soil DCF via their control over chemoautotrophic microbial communities and carbon fixation pathways. Collectively, this study provides a profound understanding of the feedback mechanisms of DCF to global warming in coastal wetlands, which helps to reduce the uncertainty for future climate change prediction and offers an important scientific basis for future carbon mitigation policies.

## Supplementary Material

Supplementary_Information_wrae138

## Data Availability

All sequence data and sample information are available at National Center for Biotechnology Information (NCBI) Sequence Read Archive (SRA) database under BioProject accession number PRJNA1058218 (https://www.ncbi.nlm.nih.gov/bioproject/PRJNA1058218). All data needed to evaluate the conclusions in the paper are present in the paper and/or the Supplementary Materials. The pipeline to process metagenomic samples is available at Figshare (https://doi.org/10.6084/m9.figshare.23768061).
