# Supplementary material for: Four years of climate warming reduced dark carbon fixation in coastal wetlands
Source: ISME J. 2024 Jul 25;18(1):wrae138. doi: 10.1093/ismejo/wrae138 (PMC11308615; doi:10.1093/ismejo/wrae138)
Supplement: Supplementary_Information_wrae138 [file supplementary_information_wrae138.docx]

**Four years of climate warming reduced dark carbon fixation in coastal wetlands**

*Bolin Liu^1,2^†, Lin Qi^1^†, Yanling Zheng^1,2^*, Chao Zhang^1^*, Jie Zhou^2^, Zhirui An^2^, Bin Wang^2^, Zhuke Lin^1^, Cheng Yao^1^, Yixuan Wang^1^, Guoyu Yin^1^, Hongpo Dong^2^, Xiaofei Li^2^, Xia Liang^2^, Ping Han^1^, Min Liu^1^, Guosen Zhang^2^, Ying Cui^2^, Lijun Hou^2^**

^1^Key Laboratory of Geographic Information Science of the Ministry of Education, School of Geographic Sciences, East China Normal University, 500 Dongchuan Road, Shanghai 200241, China

^2^State Key Laboratory of Estuarine and Coastal Research, Yangtze Delta Estuarine Wetland Ecosystem Observation and Research Station, East China Normal University, 500 Dongchuan Road, Shanghai 200241, China

†These authors contributed equally to this work.

*Corresponding authors: Yanling Zheng (ylzheng@geo.ecnu.edu.cn), Chao Zhang (zhangchao@geo.ecnu.edu.cn), and Lijun Hou (ljhou@sklec.ecnu.edu.cn)

Tel: 86-021-54836040

Fax: 86-021-54836458

# Supplementary Methods

## Measurement of dark carbon fixation (DCF) rates

DCF rates of soil were determined using the ^13^C-CO_2_ labelling method [1-3]. Briefly, soil sample (20 g) was placed into a 120 ml serum bottle. The bottles were sealed with butyl rubber septa and aluminum caps and pre-incubated in the dark at near *in situ* temperature for 48 h. Following the pre-incubation period, all bottles were opened, and the headspace was flushed with synthetic air (75% N_2_ and 25% O_2_ for Ah layer; 100% N_2_ for Bh layer) for 5 minutes. Subsequently, the bottles were sealed immediately. 5% (v/v) ^13^CO_2_ and ^12^CO_2_ were added to the experimental and control incubations using syringes, respectively. The samples were incubated in the dark at near *in situ* temperature for 8 days (Fig. S2). During the incubation, the headspace of the bottles was flushed with synthetic air every 2 days, and the ^13^CO_2_ or ^12^CO_2_ were re-injected.

After incubation, the samples were freeze-dried and ground using a mortar. TOC and δ^13^C values of the samples were measured after acidification with 1 M HCl. The δ^13^C values were determined using DeltaPlus XP isotope ratio mass spectrometer (IRMS) (Thermo Fisher Scientific, CA, USA). DCF rates of the soils were calculated from the isotope ratio of the ^13^CO_2_ and ^12^CO_2_ incubations. The stable isotope abundance was represented as δ-values (‰) relative to the standard V-PDB, which was calculated as:

$$\delta{}^{13}C=\left( \frac{R_{sample}}{R_{standard}}-1 \right)\times{10}^{3} (1)$$

where R_standard_ is 0.0111802, R_sample_ is the ratio of ^13^C to ^12^C.

^13^C atom % was calculated as:

$$F=\frac{R}{R+1} (2)$$

DCF rates were calculated as:

$$DCF=\frac{\mathrm{TOC}}{t}\times\left( F_{a}-F_{b} \right) (3)$$

where TOC is the total organic carbon in soil, t is the incubation time, F_a_ and F_b_ were the ^13^C atom % of the ^13^CO_2_ and ^12^CO_2_ incubations, respectively.

## Measurement of carbon mineralization (CM) rates

Soil organic carbon mineralization (CM) was evaluated by CO_2_ emissions through incubation experiments [4]. Soil sample (30 g) was placed into a 250 ml serum bottle and incubated in the dark at near *in situ* temperature for 15 days. The CO_2_ released was determined by gas chromatography (GC-2014, Shimadzu, Kyoto, Japan) at 1, 2, 3, 7, 10, and 15 days after incubation. During the incubation, CO_2_ emission was used to calculate CM rates.

## Measurement of the biomass of *Phragmites australis*

Samples of plants and soil cores were collected to measure above- and below-ground biomass [5] of *Phragmites australis* during April (spring), July (summer), Autumn (autumn), and January (winter). Four 30 cm × 30 cm square plots of above-ground plants were harvested and thoroughly washed with clean water. For the estimation of below-ground biomass, five soil cores were randomly selected at each sampling site and extracted using a steel corer with a diameter of 7 cm and length of 60 cm. Below-ground root samples were rinsed in running water using a 0.5 mm mesh sieve to remove the soil. Following cleaning, the above-ground plants and below-ground root were oven-dried at 105°C for 2 h to eliminate enzymatic activity, and subsequently dried at 60°C until a constant weight was achieved. Finally, the dry weight of the samples was measured to calculate the above- and below-ground biomass.

## Statistical analysis

Linear mixed-effects models (LMMs) were employed to establish connections between individual environmental variables and the dark carbon fixation (DCF) process along with its associated chemoautotrophic microbial communities. Sampling season and layer were treated as random intercept effects. The *MuMIn* R package’s 'r.squaredGLMM' function was utilized to compute the marginal coefficients of determination, which indicate the amount of variance explained by the fixed effect in the LMMs [6]. In order to distinguish the direct and indirect effects of environmental drivers, as well as the diversity and abundance of chemoautotrophic microbial communities on DCF rates, we employed structural equation modelling (SEM) with the *lavaan* R package [7]. Prior to the SEM analysis, a Pearson’s correlation analysis was conducted to select subsets of environmental variables with the least amount of correlation. These variables were standardized using 'scale' function within different seasons.

# Supplementary Figures


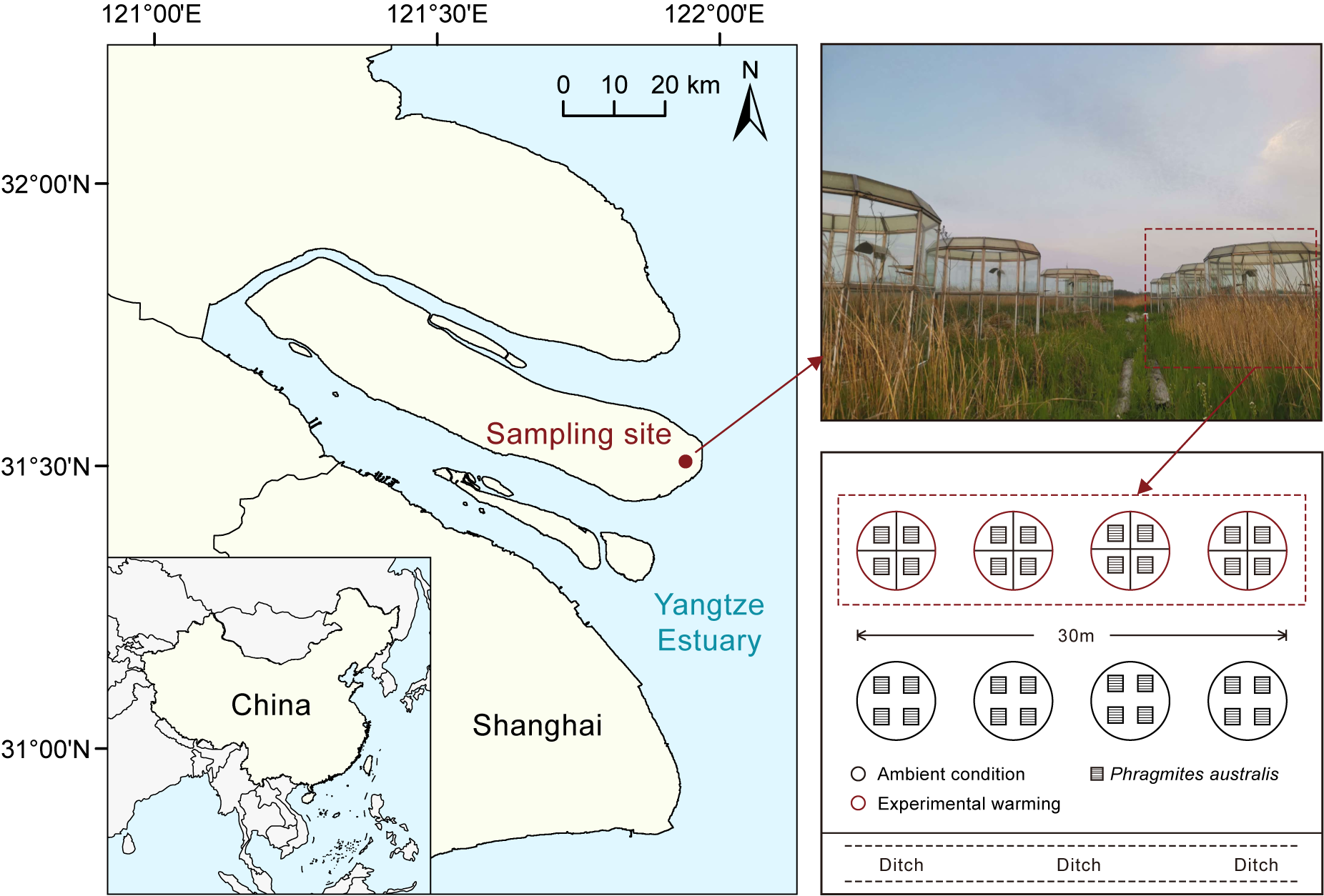


Fig. S1 The geographical location of the Yangtze Estuary Wetland and a schematic map of the field experimental treatments.


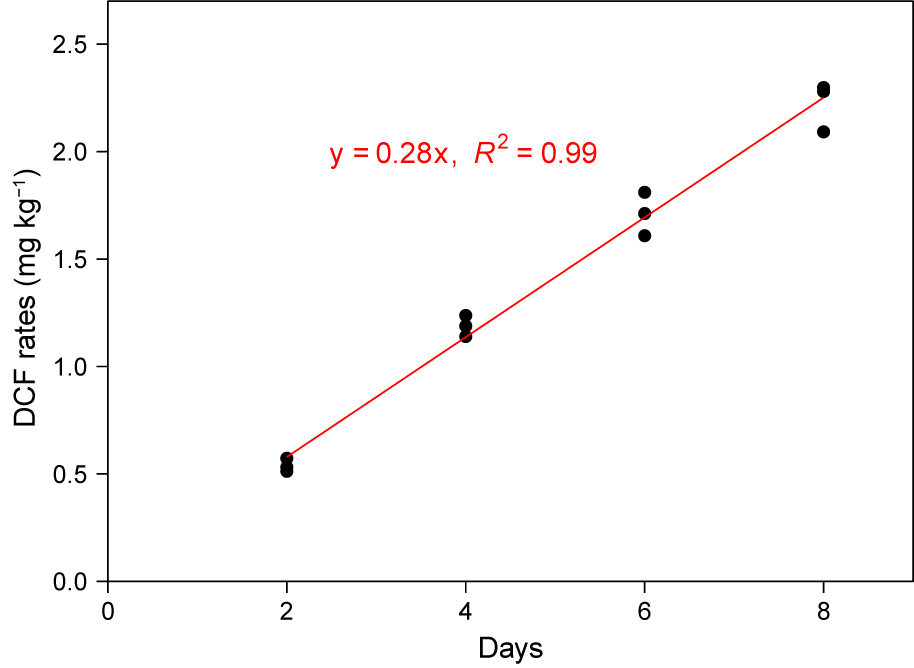


Fig. S2 Time series of the uptake of ^13^C by microorganisms.


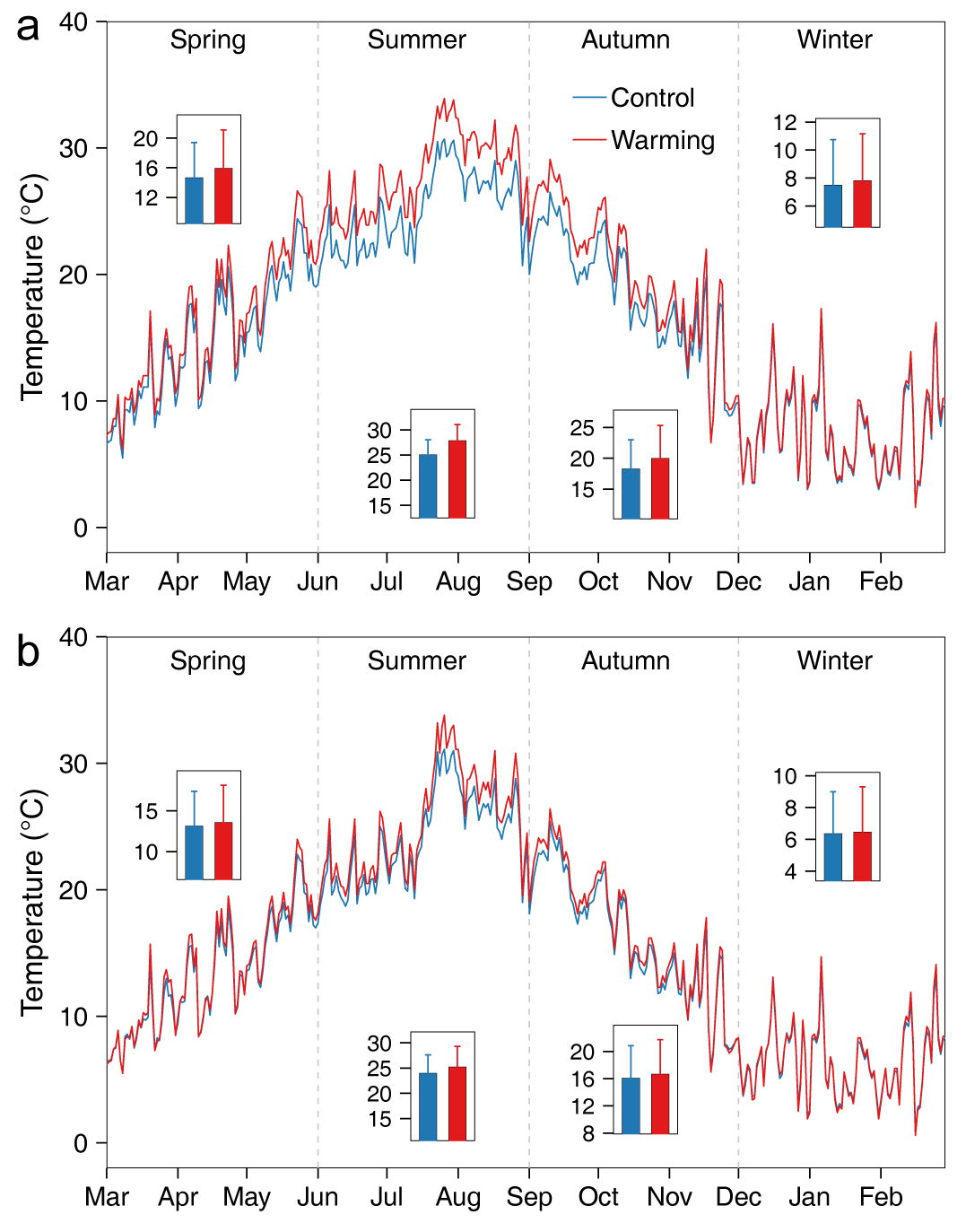


Fig. S3 Seasonal variation of air temperature (a) and soil temperature (b) in the control (blue) and warming (red) plots. The bars represent the average temperature of different seasons.


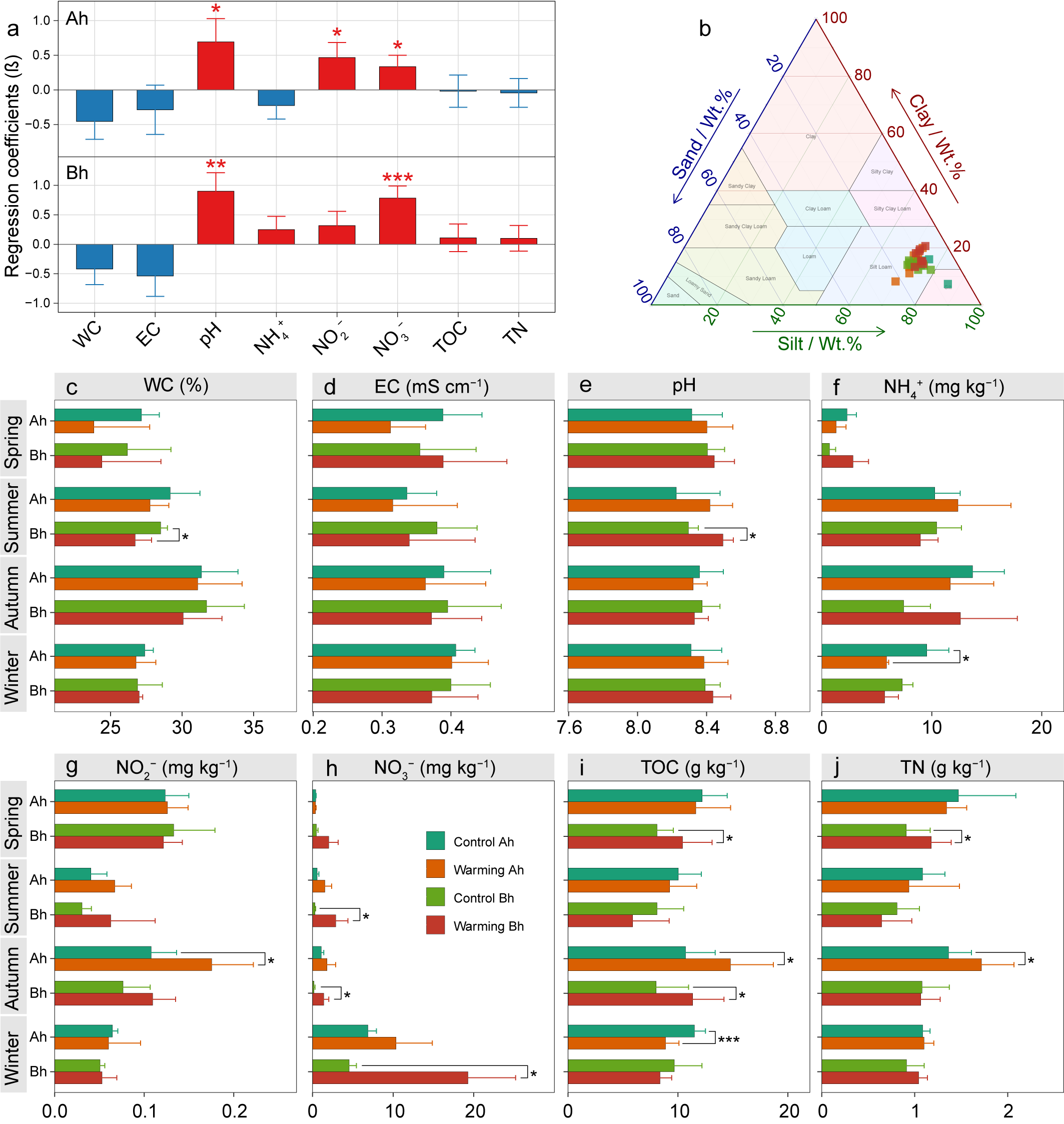


Fig. S4 Environmental physicochemical parameters of coastal wetland soils in response to climate warming. (a), The regression coefficients (β) of warming on the DCF rates were determined through linear mixed-effects models (LMMs). Ah and Bh represent surface (0 - 5 cm) and subsurface (5 - 10 cm) layers of the soil, respectively. (b), Grain size of coastal wetland soil samples. (c-j), Effects of warming on water content (WC), EC, pH, NH_4_^+^, NO_2_^−^, NO_3_^−^, TOC and TN of coastal wetland soils across four seasons. Data are presented as mean and standard deviation (SD) (n=12). The asterisk above the column denotes significant differences between control and warming treatments (*P* < 0.05).


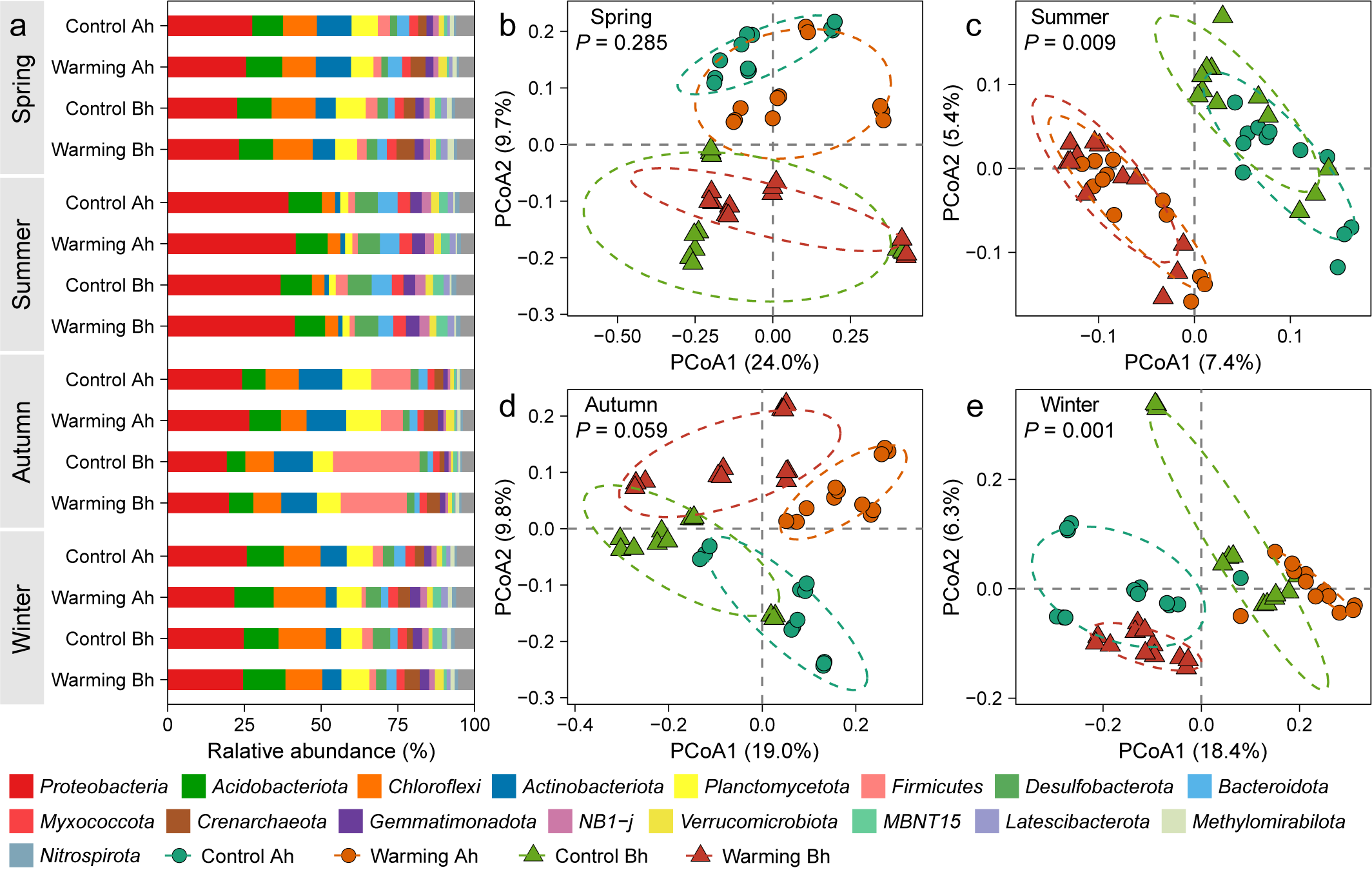


Fig. S5 Effects of warming on microbial community composition in coastal wetland soils. (a), Relative abundance of most frequent microbial phyla in soils based on the bacterial and archaeal 16S rRNA gene sequences. (b-e), PCoA analyses with Bray-Curtis dissimilarity of 16S rRNA sequences in different seasons. Ah and Bh represent surface (0 - 5 cm) and subsurface (5 - 10 cm) layers of the soil. The effects were analyzed using PERMANOVA statistical tests with 999 permutations.


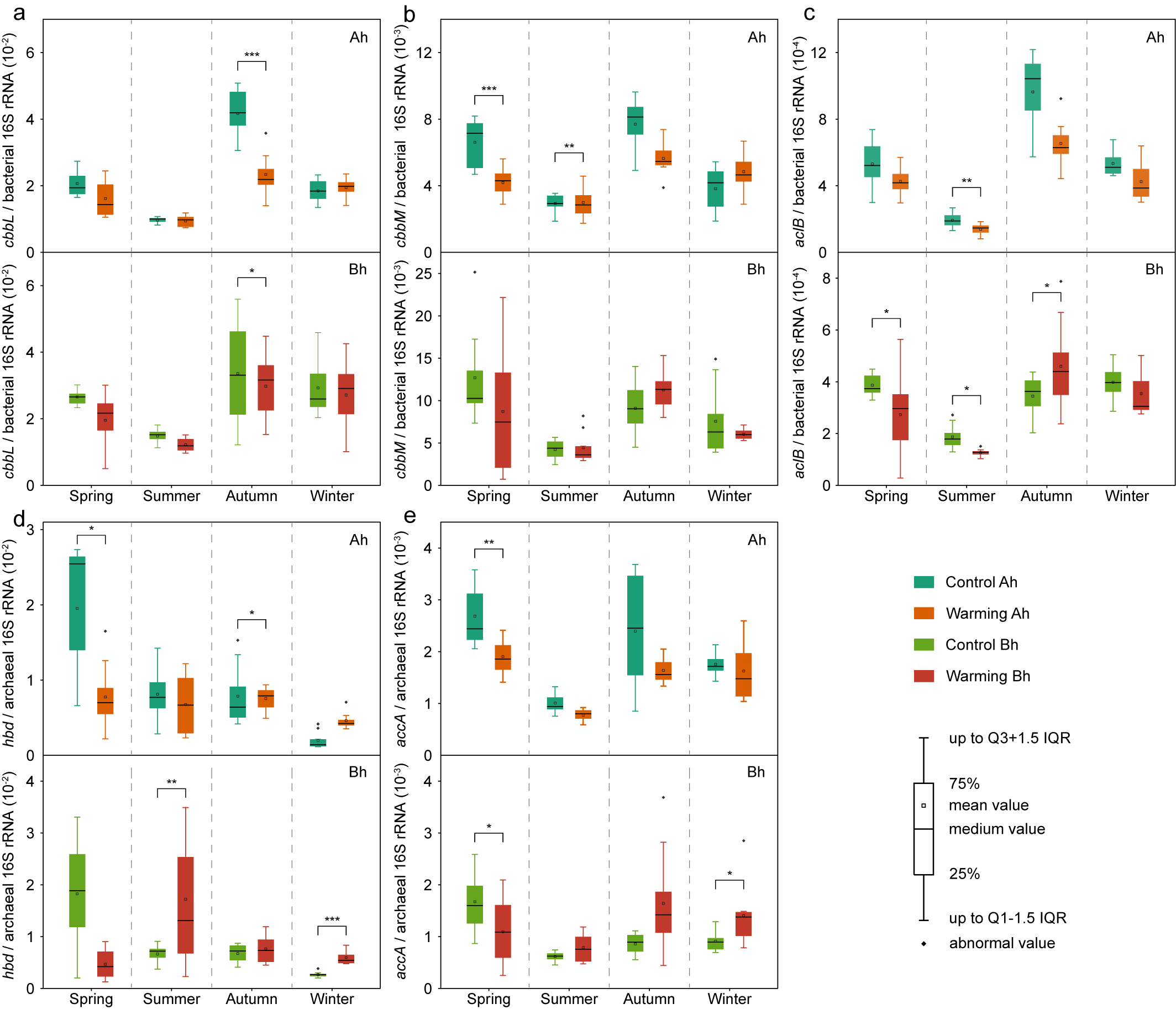


Fig. S6 Abundances of *cbbL* (a), *cbbM* (b), *aclB* (c), *hbd* (d) and *accA* (e) genes in coastal wetland soils. The abundances of *cbbL*, *cbbM*, and *aclB* genes were normalized based on the copies of bacterial 16S rRNA gene, while the abundances of *hbd* and *accA* genes were normalized based on the copies of archaeal 16S rRNA gene. Ah and Bh represent surface (0 - 5 cm) and subsurface (5 - 10 cm) layers of the soil, respectively. Boxes represent the interquartile range (IQR) between the first and third quartiles (25th and 75th percentiles, respectively), and the horizontal line inside the box defines the median. Whiskers represent the lowest and highest values within 1.5 times the IQR from the first and third quartiles, respectively. Statistical significance was determined using either Student's t-test or Mann-Whitney test. The asterisk above the column denotes significant differences between control and warming treatments (**P* < 0.05, ***P* < 0.01, and ****P* < 0.001).


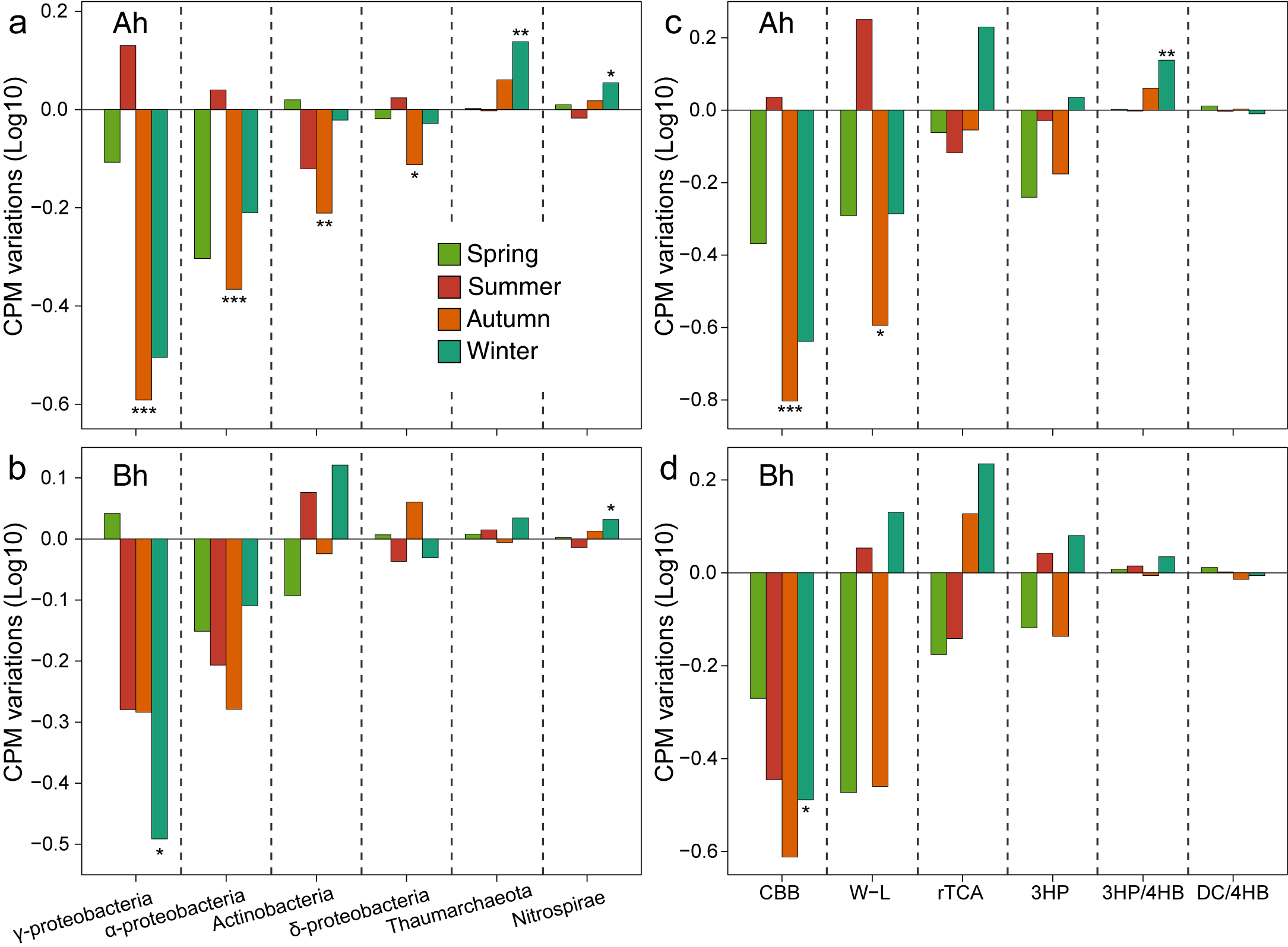


Fig. S7 Effects of warming on the relative abundance of chemoautotrophic community and their carbon fixation pathways in different seasons. The CPM variations between control and warming groups were normalized using the transformation [log_10_(|CPM variation|+1) × (1 or -1)]. Ah and Bh represent surface (0 - 5 cm) and subsurface (5 - 10 cm) layers of the soil, respectively. Statistical significance was determined using either Student's t-test or Mann-Whitney test. The asterisk above the column denotes significant differences between control and warming treatments (*P* < 0.05).


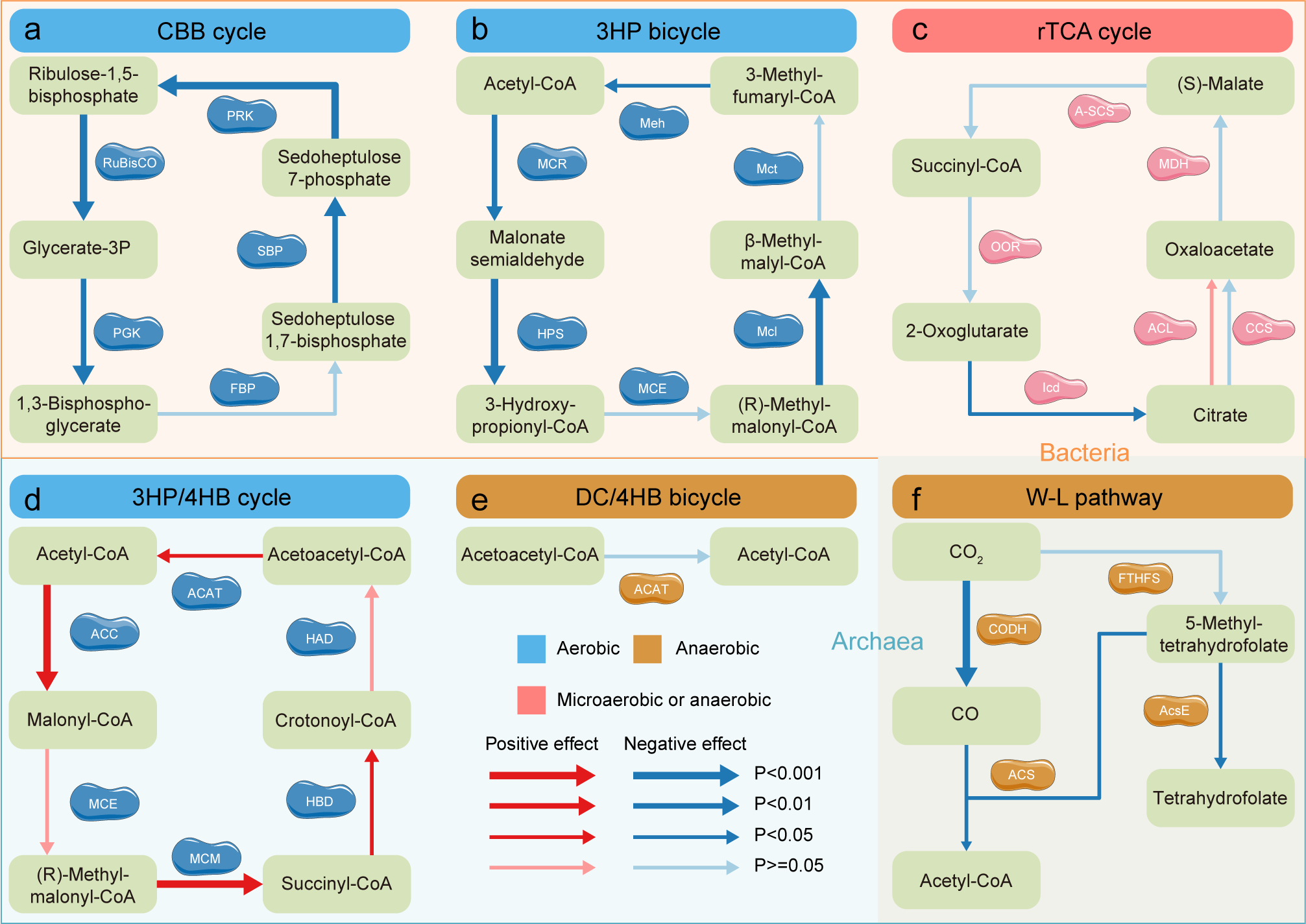


Fig. S8 Different responses of various carbon fixation pathways in coastal wetland soils under simulated warming. Effects were determined using linear mixed-effects models (LMMs), in which seasons, plots and layers were considered as random intercept effects. The background color of each subgraph title indicates oxygen conditions required for the pathways: blue for aerobic, brown for anaerobic, and pink for microaerobic or anaerobic.


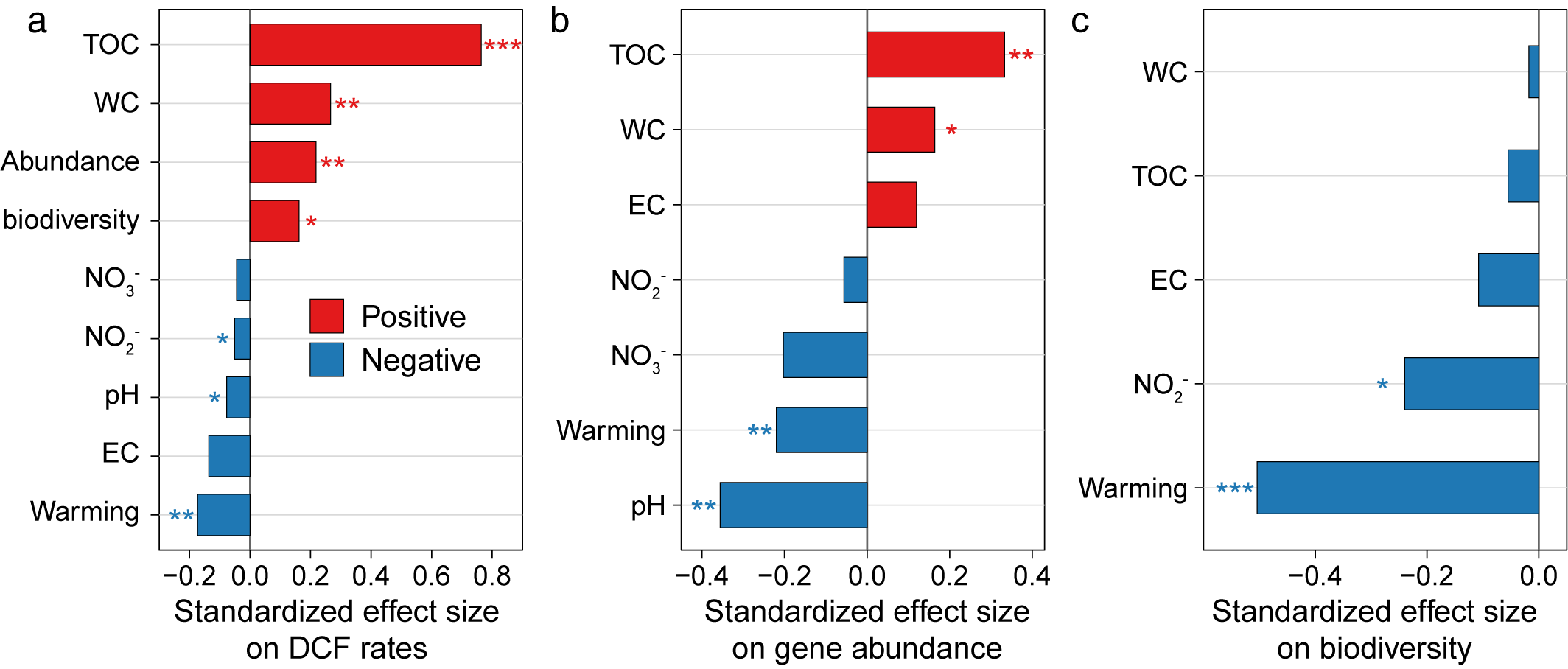


Fig. S9 Standardized effects for direct and indirect contributions of environmental parameters on DCF rates (a), as well as abundance (b) and biodiversity (c) of chemoautotrophic microbial community in Structural equation models (SEMs) analysis. Significance levels are denoted by asterisks (**P* < 0.05, ***P* < 0.01, and ****P* < 0.001).


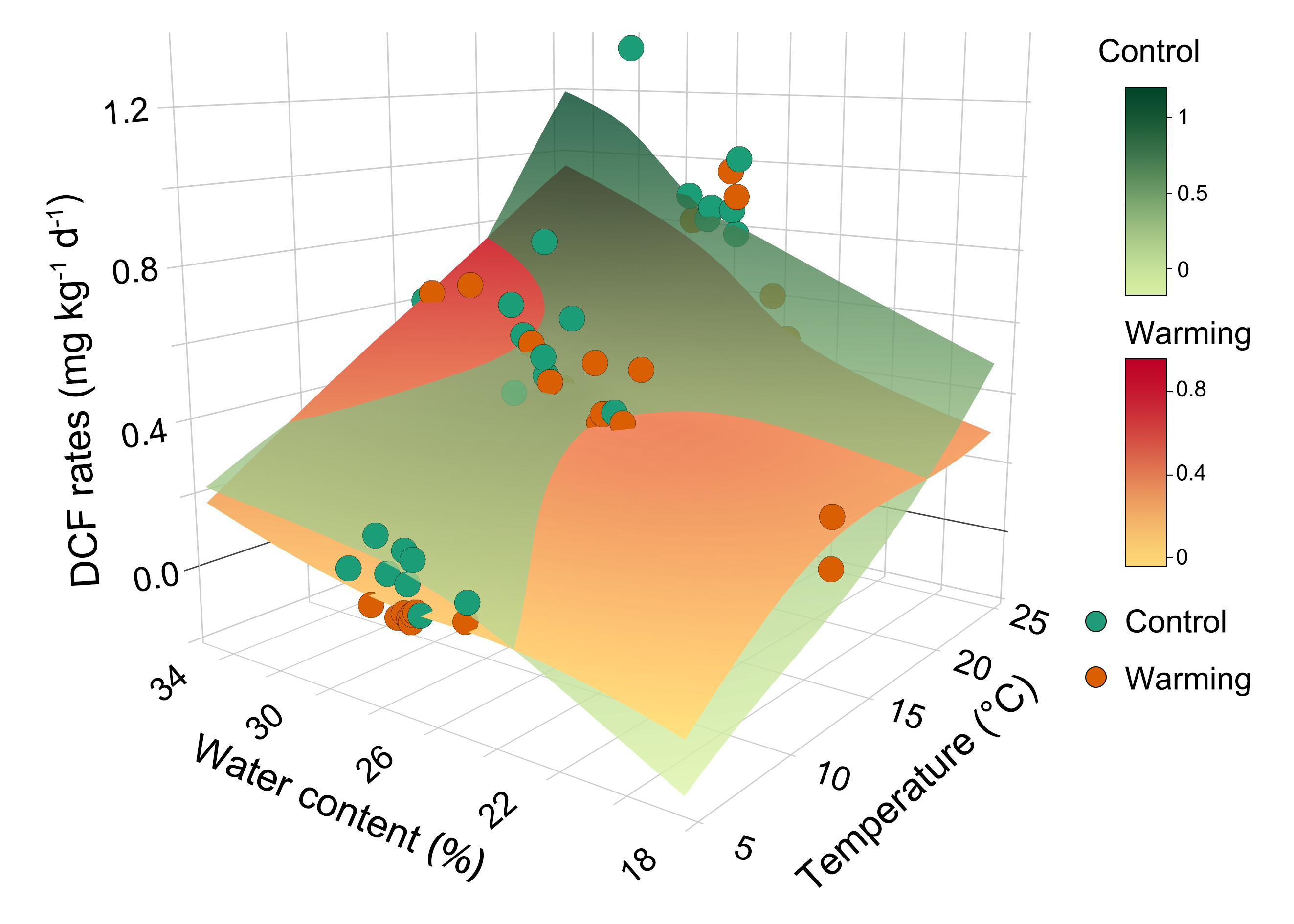


Fig. S10 Relationship between DCF rates and environmental variables including temperature and water content based on nonlinear regression with generalized additive model (GAM). The simulated warming treatment and ambient control plots were represented by the color red and green, respectively.


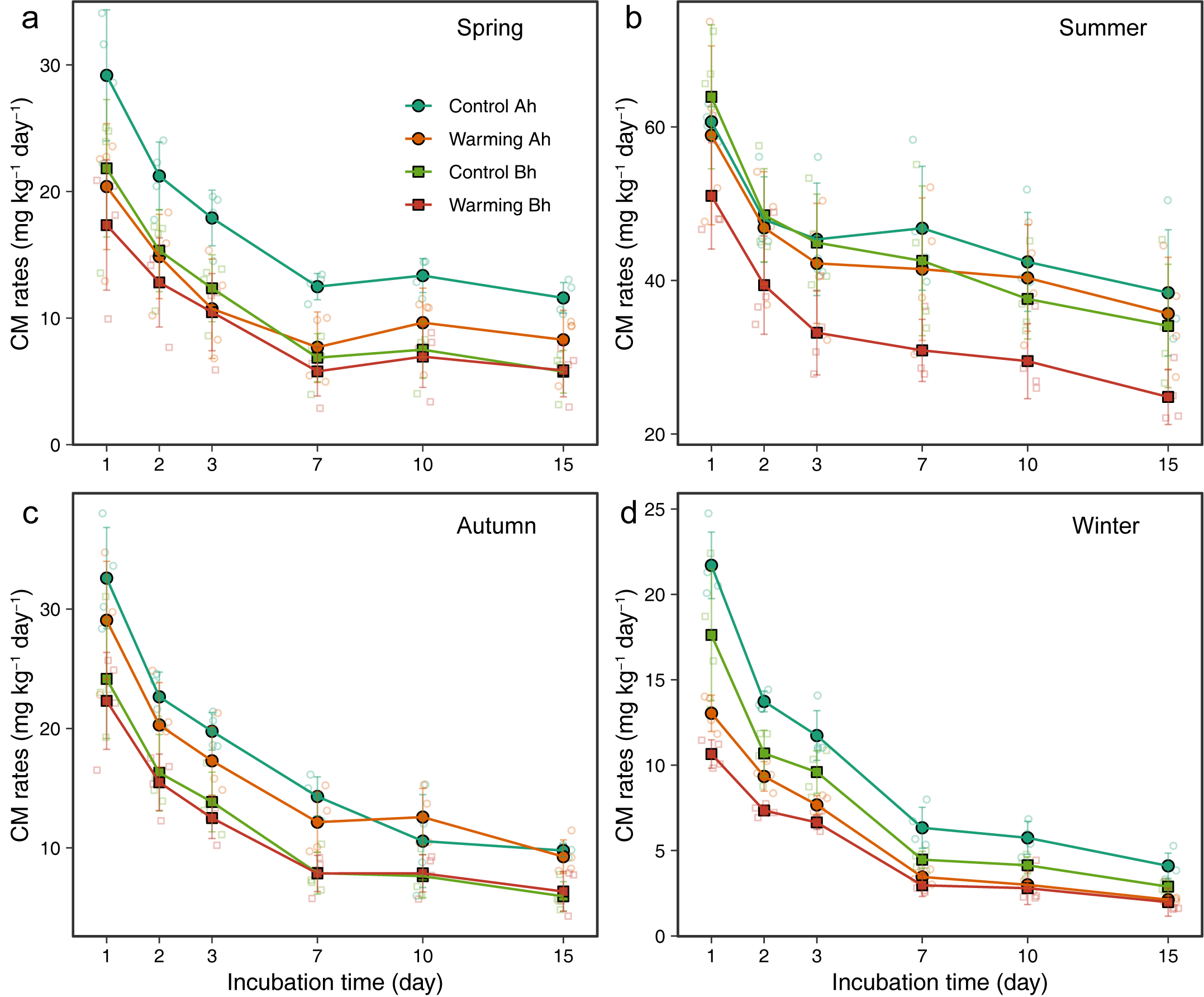


Fig. S11 Effect of warming on carbon mineralization (CM) rates in coastal wetland soils during different seasons. Ah and Bh represent surface (0 - 5 cm) and subsurface (5 - 10 cm) layers of the soil, respectively. Error bar denotes the standard deviation (SD) (n=4).


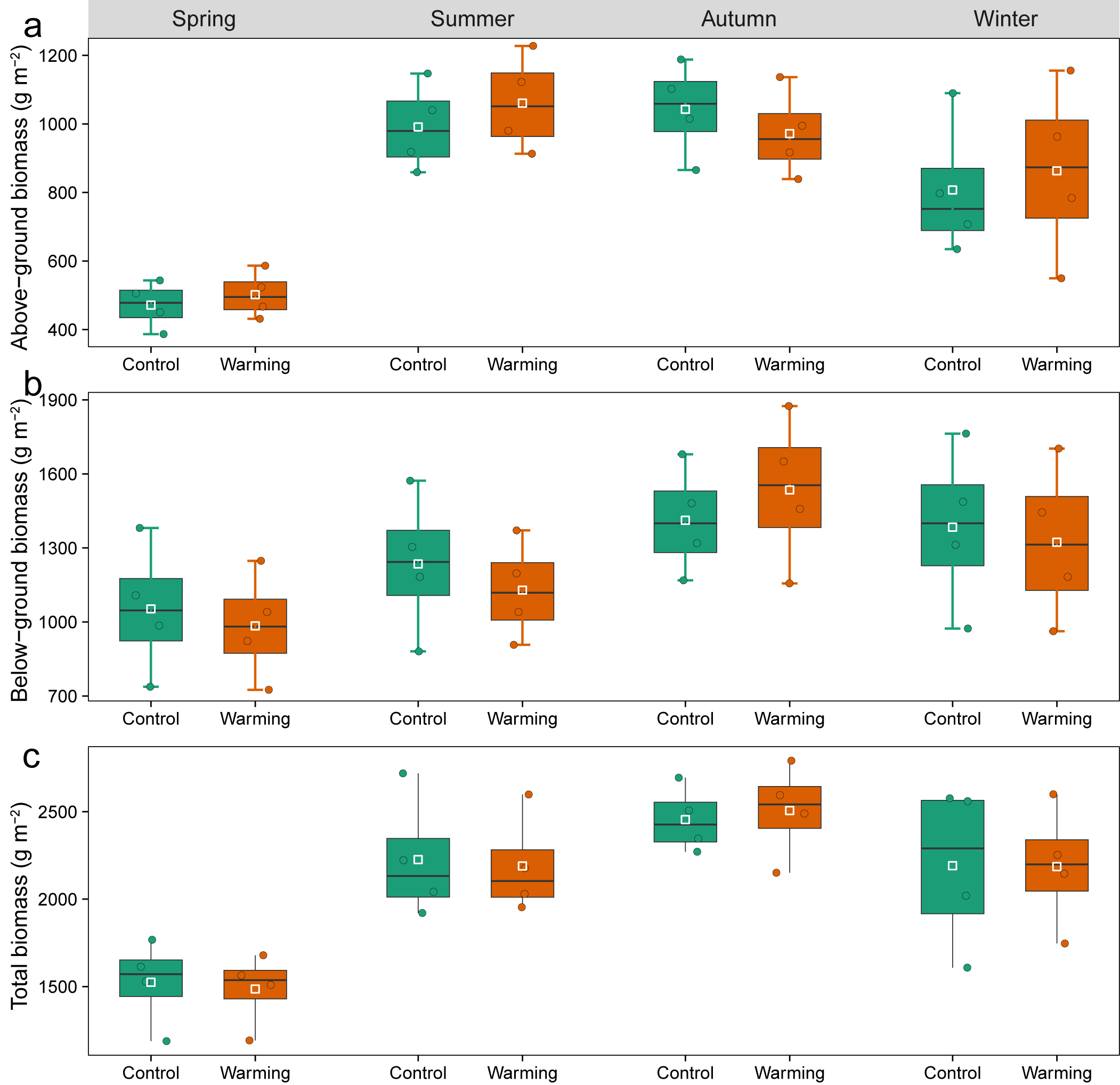


Fig. S12 Seasonal variation of biomass of *Phragmites australis* in the control and warming plots in coastal wetland soils. (a), Above-ground biomass. (b), Below-ground biomass. (c), Total biomass. Boxes represent the interquartile range (IQR) between the first and third quartiles (25th and 75th percentiles, respectively), and the horizontal line inside the box defines the median (n = 4). Whiskers represent the lowest and highest values within 1.5 times the IQR from the first and third quartiles, respectively.


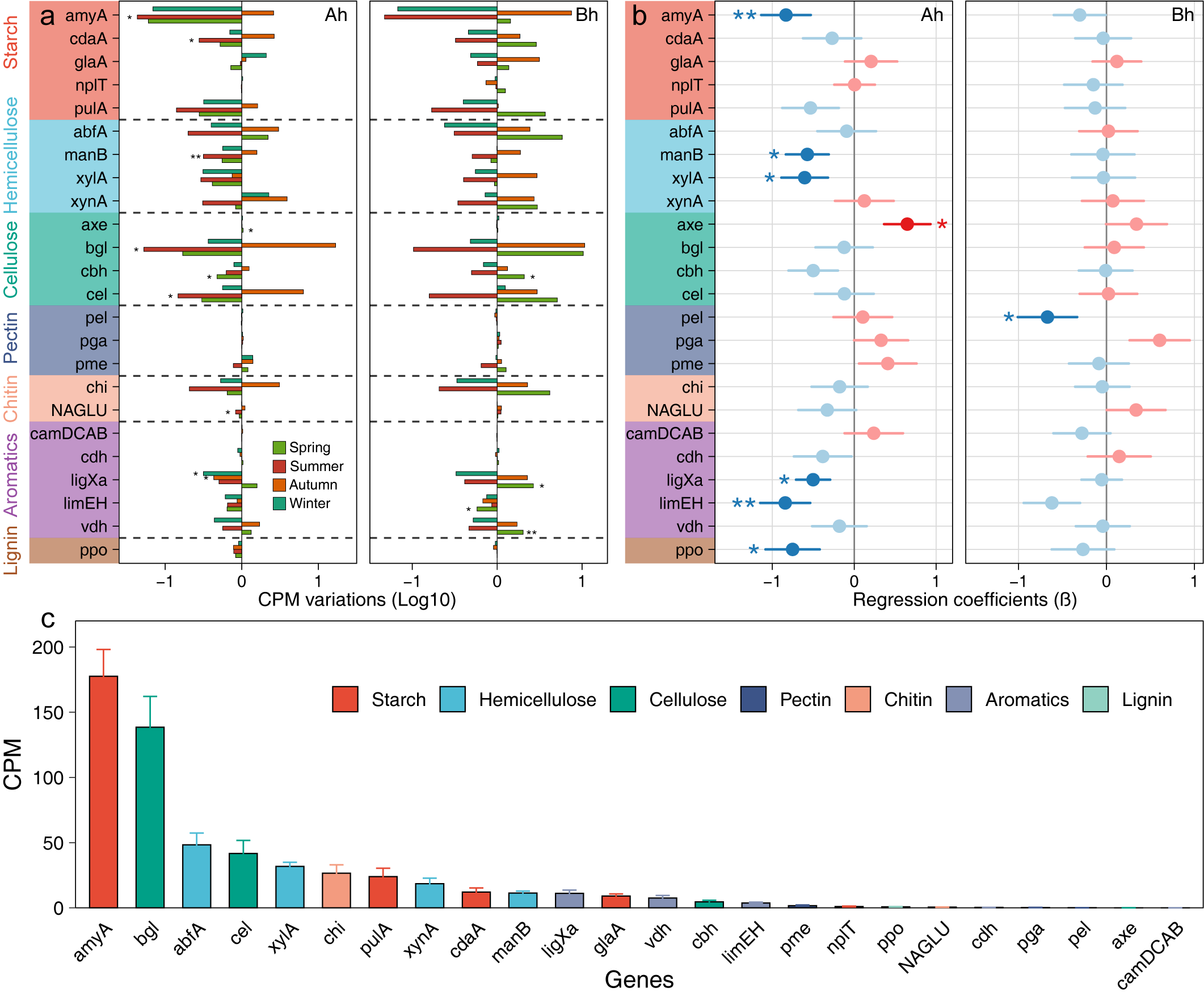


Fig. S13 Effects of warming on enzymes associated with heterotrophic respiration in coastal wetland soils. (a-b), Effects of warming on the normalized abundance of enzymes associated with heterotrophic respiration, determined through t test and linear mixed-effects models (LMMs) analyses. The CPM variations between control and warming groups were normalized using the transformation [log_10_(|CPM variation|+1) × (1 or -1)]. Ah and Bh represent surface (0 - 5 cm) and subsurface (5 - 10 cm) layers of the soil, respectively. The asterisk above the column denotes significant differences between control and warming treatments (*P* < 0.05). (c), Normalized abundance of enzymes associated with heterotrophic soil respiration based on the metagenome analysis. Error bar denotes the standard deviation (SD) (n=4).


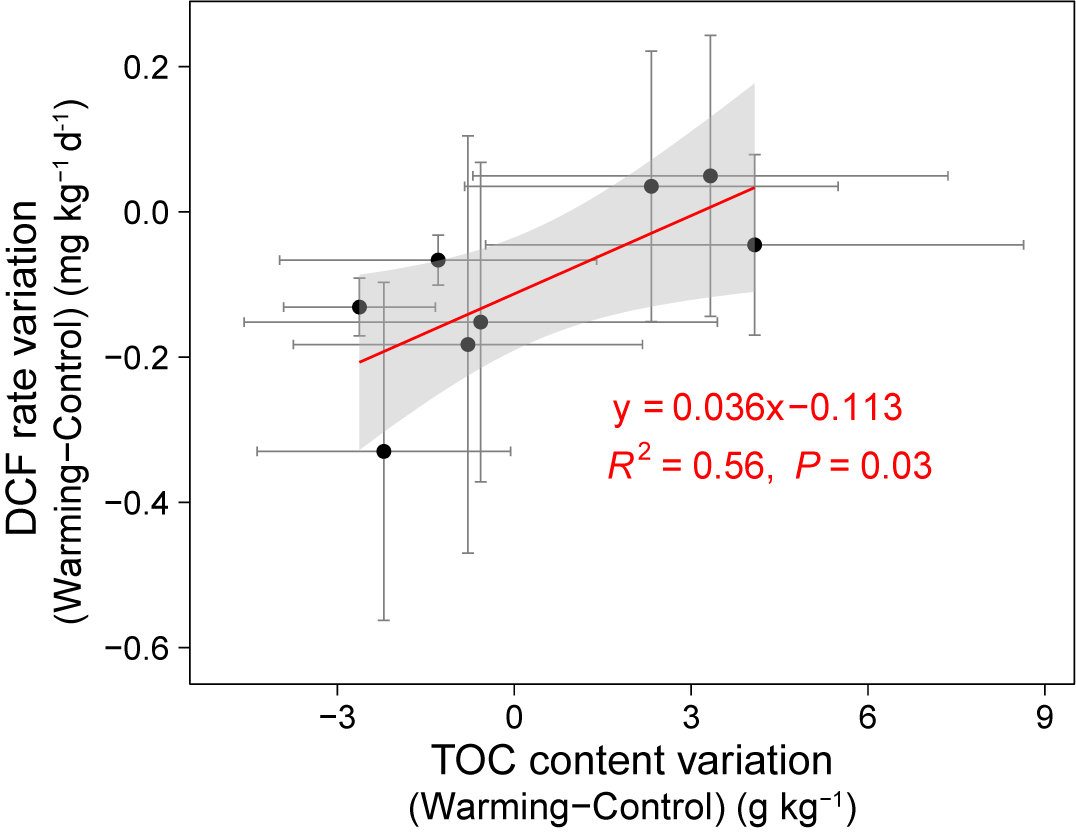


Fig. S14 Relationship between the variations (Warming-Control) in both DCF rates and TOC content under simulated warming. Horizonal and vertical error bars represent the standard deviation (SD) (n=4) for TOC content and DCF rates, respectively.

# Supplementary Tables

Table S1 Primers and thermal cycling conditions for qPCR.

| Target genes | Primer pairs (5' - 3') | Tm | References |
| --- | --- | --- | --- |
| *cbbL* gene | cbbL_K2f: ACCAYCAAGCCSAAGCTSGG  cbbL_V2r: GCCTTCSAGCTTGCCSACCRC | 63°C | [8] |
| *cbbM* gene | cbbM-f: GGCACCATCATCAAGCCCAAG  cbbM-r: TCTTGCCGTAGCCCATGGTGC | 57°C | [9] |
| *aclB* gene | aclB_402F: GGTGAAACGCCTCTGCTT  aclB_907R: ATACTTCCACGGCCCAAT | 55°C | [10] |
| *hbd* gene | hbd-f: GACTGATCCWAAAGGDGAYAGAAG  hbd-r: CCYTTARCATCTGCWGGAATTGC | 56°C | [11] |
| *accA* gene | Crena_529F: GCWATGACWGAYTTTGTYRTAATG  Crena_981R: TGGWTKRYTTGCAAYTATWCC | 50°C | [12] |
| Bacterial  16S rRNA gene | 341F: CCTACGGGAGGCAGCAG  518R: ATTACCGCGGCTGCTGG | 59°C | [13] |
| Archaeal  16S rRNA gene | Arch-967F: AATTGGCGGGGGAGCAC  Arch-1060R: GGCCATGCACCWCCTCTC | 56°C | [14] |

Table S2 Library size for all metagenomes constructed.

| Sample | Season | Layer | Treat | Depth (Gbp) | High-quality ReadsNum | High-quality BasesNum |
| --- | --- | --- | --- | --- | --- | --- |
| S-CK1S | Spring | Ah | Control | 17.1 | 111709364 | 16838624826 |
| S-CK2S | Spring | Ah | Control | 16.3 | 106473534 | 16036711249 |
| S-CK3S | Spring | Ah | Control | 19.8 | 129525142 | 19498839182 |
| S-CK4S | Spring | Ah | Control | 15.5 | 101395958 | 15264728069 |
| S-SW1S | Spring | Ah | Warming | 17.3 | 112827344 | 17011375567 |
| S-SW2S | Spring | Ah | Warming | 16.4 | 106673080 | 16073039919 |
| S-SW3S | Spring | Ah | Warming | 19.2 | 125628916 | 18917605420 |
| S-SW4S | Spring | Ah | Warming | 16.8 | 109291284 | 16461133026 |
| S-CK1B | Spring | Bh | Control | 16.5 | 107451406 | 16196102022 |
| S-CK2B | Spring | Bh | Control | 17.1 | 112015882 | 16876764378 |
| S-CK3B | Spring | Bh | Control | 18.0 | 117535332 | 17700207744 |
| S-CK4B | Spring | Bh | Control | 15.8 | 102970910 | 15525996982 |
| S-SW1B | Spring | Bh | Warming | 18.4 | 120269566 | 18120172837 |
| S-SW2B | Spring | Bh | Warming | 17.4 | 113824010 | 17143180653 |
| S-SW3B | Spring | Bh | Warming | 17.5 | 114639250 | 17263942175 |
| S-SW4B | Spring | Bh | Warming | 16.6 | 108313710 | 16306903000 |
| X-CK1S | Summer | Ah | Control | 12.7 | 82509404 | 12430620821 |
| X-CK2S | Summer | Ah | Control | 10.8 | 70292986 | 10593810795 |
| X-CK3S | Summer | Ah | Control | 13.8 | 89704284 | 13515865759 |
| X-CK4S | Summer | Ah | Control | 12.8 | 82763110 | 12465612529 |
| X-SW1S | Summer | Ah | Warming | 12.6 | 81938404 | 12343282048 |
| X-SW2S | Summer | Ah | Warming | 11.5 | 74982820 | 11301559492 |
| X-SW3S | Summer | Ah | Warming | 12.1 | 78836564 | 11880266100 |
| X-SW4S | Summer | Ah | Warming | 13.0 | 83952058 | 12646166244 |
| X-CK1B | Summer | Bh | Control | 15.2 | 98922016 | 14902760915 |
| X-CK2B | Summer | Bh | Control | 11.9 | 77348610 | 11655107995 |
| X-CK3B | Summer | Bh | Control | 12.1 | 78584444 | 11836279216 |
| X-CK4B | Summer | Bh | Control | 10.7 | 69646176 | 10488272560 |
| X-SW1B | Summer | Bh | Warming | 12.5 | 81537852 | 12287692518 |
| X-SW2B | Summer | Bh | Warming | 12.0 | 78305964 | 11801358000 |
| X-SW3B | Summer | Bh | Warming | 11.9 | 76965310 | 11595694871 |
| X-SW4B | Summer | Bh | Warming | 11.9 | 77358148 | 11656733401 |

**Table S2 (continued).**

| Sample | Season | Layer | Treat | Depth (Gb) | High-quality ReadsNum | High-quality BasesNum |
| --- | --- | --- | --- | --- | --- | --- |
| A-CK1S | Autumn | Ah | Control | 19.9 | 129884666 | 19561011588 |
| A-CK2S | Autumn | Ah | Control | 18.8 | 122453692 | 18442965056 |
| A-CK3S | Autumn | Ah | Control | 21.3 | 138743680 | 20911097427 |
| A-CK4S | Autumn | Ah | Control | 18.6 | 121866200 | 18364546858 |
| A-SW1S | Autumn | Ah | Warming | 18.5 | 120727342 | 18169120446 |
| A-SW2S | Autumn | Ah | Warming | 17.3 | 112702874 | 16986709864 |
| A-SW3S | Autumn | Ah | Warming | 17.1 | 111255806 | 16764391219 |
| A-SW4S | Autumn | Ah | Warming | 17.9 | 116442104 | 17543981943 |
| A-CK1B | Autumn | Bh | Control | 23.7 | 154103828 | 23209610287 |
| A-CK2B | Autumn | Bh | Control | 19.2 | 125477568 | 18915272372 |
| A-CK3B | Autumn | Bh | Control | 20.3 | 132435010 | 19959575989 |
| A-CK4B | Autumn | Bh | Control | 19.2 | 125424586 | 18894889757 |
| A-SW1B | Autumn | Bh | Warming | 18.0 | 117271906 | 17672225686 |
| A-SW2B | Autumn | Bh | Warming | 16.9 | 109988440 | 16579071409 |
| A-SW3B | Autumn | Bh | Warming | 16.4 | 106728404 | 16084360639 |
| A-SW4B | Autumn | Bh | Warming | 15.9 | 103807276 | 15644198060 |
| W-CK1S | Winter | Ah | Control | 16.5 | 107874604 | 16268455229 |
| W-CK2S | Winter | Ah | Control | 18.1 | 118240388 | 17818082388 |
| W-CK3S | Winter | Ah | Control | 17.8 | 115947818 | 17477391993 |
| W-CK4S | Winter | Ah | Control | 19.5 | 126454928 | 19017754792 |
| W-SW1S | Winter | Ah | Warming | 18.0 | 117320696 | 17683038793 |
| W-SW2S | Winter | Ah | Warming | 19.6 | 127820136 | 19268766667 |
| W-SW3S | Winter | Ah | Warming | 21.5 | 139837208 | 21077116994 |
| W-SW4S | Winter | Ah | Warming | 18.3 | 119774974 | 18055658436 |
| W-CK1B | Winter | Bh | Control | 20.5 | 133505588 | 20124364486 |
| W-CK2B | Winter | Bh | Control | 21.4 | 139032256 | 20953382276 |
| W-CK3B | Winter | Bh | Control | 20.8 | 135705100 | 20456913967 |
| W-CK4B | Winter | Bh | Control | 20.4 | 133020730 | 20046468616 |
| W-SW1B | Winter | Bh | Warming | 20.6 | 134856046 | 20321801292 |
| W-SW2B | Winter | Bh | Warming | 21.4 | 138799170 | 20919695610 |
| W-SW3B | Winter | Bh | Warming | 21.2 | 138048764 | 20794496916 |
| W-SW4B | Winter | Bh | Warming | 16.5 | 107530366 | 16201040848 |

Table S3 Maker enzymes used to identify chemoautotrophs utilizing various carbon fixation pathways.

| Carbon fixation pathway | Key enzymes | KO |
| --- | --- | --- |
| Calvin-Benson-Bassham (CBB) cycle | **Ribulose-1,5-bisphosphate carboxylase/oxygenase (RuBisCO)*** | **K01601*** |
|  |  | K01602 |
|  | Phosphoglycerate kinase (PGK) | K00927 |
|  | Fructose-bisphosphate aldolase (FBP) | K01623 |
|  |  | K01624 |
|  |  | K11645 |
|  | Sedoheptulose 1,7-bisphosphatase (SBP) | K11532 |
|  |  | K01086 |
|  |  | K01100 |
|  | Phosphoribulokinase (PRK) | K00855 |
| Reductive tricarboxylic acid (rTCA) cycle | 2-oxoglutarate synthase (OOR) | K00174 |
|  |  | K00175 |
|  |  | K00176 |
|  |  | K00177 |
|  | Isocitrate dehydrogenase (Icd) | K00031 |
|  | **ATP citrate lyase (ACL)*** | K15230 |
|  |  | **K15231*** |
|  |  | K01648 |
|  | **Citryl-CoA synthetase (CCS)*** | **K15232*** |
|  |  | K15233 |
|  | Malate dehydrogenase (MDH) | K00024 |
|  | Succinate-CoA ligase (ADP-forming) (A-SCS) | K01902 |
|  |  | K01903 |
| 3-hydroxypropionate bicycle (3HP) cycle | Malonyl-CoA reductase (MCR) | K14468 |
|  | 3-hydroxypropionyl-CoA synthase (HPS) | K14469 |
|  | Methylmalonyl-CoA epimerase (MCE) | K05606 |
|  | Malyl-CoA lyase (Mcl) | K08691 |
|  | **2-methylfumaryl-CoA isomerase (Mct)*** | **K14470*** |
|  | 3-methylfumaryl-CoA hydratase (Meh) | K09709 |
| Wood-Ljungdahl (W-L) pathway | **Acetyl-CoA synthase (ACS)*** | **K14138*** |
|  |  | K00197 |
|  |  | K00194 |
|  | Carbon monoxide dehydrogenase (CODH) | K00198 |
|  |  | K00196 |
|  | Formate—tetrahydrofolate ligase (FTHFS) | K01938 |
|  | 5-methyltetrahydrofolate corrinoid/  iron sulfur protein methyltransferase (AcsE) | K15023 |

**Table S3 (continued).**

| Carbon fixation pathway | Key enzymes | KO |
| --- | --- | --- |
| 3-hydroxypropionate/4-hydroxybutyrate (3HP/4HB) cycle | Acetyl-CoA carboxylase (*Thaumarchaeota*) (ACC) | K01961 |
|  |  | K18603 |
|  |  | K18604 |
|  |  | K18605 |
|  | Methylmalonyl-CoA epimerase (*Thaumarchaeota*) (MCE) | K05606 |
|  | Methylmalonyl-CoA mutase (MCM) | K01847 |
|  |  | K01848 |
|  |  | K01849 |
|  | **4-hydroxybutyryl-CoA dehydratase (HBD)*** | **K14534*** |
|  | 3-hydroxyacyl-CoA dehydrogenase (HAD) | K15016 |
|  | Acetyl-CoA C-acetyltransferase (*Thaumarchaeota*) (ACAT) | K00626 |
| Dicarboxylate/4-hydroxybutyrate (DC/4HB) cycle | Acetyl-CoA C-acetyltransferase  (*Crenarchaeota*) (ACAT) | K00626 |

Note: The asterisks denote the key marker enzyme of different carbon fixation pathways.

Table S4 Organic carbon degradation enzymes targeting different carbon sources.

| C source | Enzymes | Abbr. | KO |
| --- | --- | --- | --- |
| Starch | Alpha-amylase | amyA | K01176 |
|  |  |  | K05343 |
|  |  |  | K07405 |
|  | Cyclomaltodextrinase | cdaA | K01208 |
|  | Glucoamylase | glaA | K01178 |
|  |  |  | K12047 |
|  | Neopullulanase | nplT | K21574 |
|  | Pullulanase | pulA | K01200 |
| Hemicellulose | Alpha-L-arabinofuranosidase | abfA | K01209 |
|  |  |  | K20844 |
|  | Beta-mannosidase | manB | K01192 |
|  | Xylose isomerase | xylA | K01805 |
|  | Xylanase | xynA | K01181 |
|  |  |  | K13465 |
| Cellulose | Acetylxylan esterase | axe | K05972 |
|  | Beta-glucosidase | bgl | K01188 |
|  |  |  | K05349 |
|  |  |  | K05350 |
|  | Cellulose 1,4-beta-cellobiosidase | cbh | K01225 |
|  |  |  | K19668 |
|  | Cellulase | cel | K01179 |
|  |  |  | K20542 |
|  |  |  | K19357 |
| Pectin | pectin lyase | pel | K01732 |
|  |  |  | K19551 |
|  | polygalacturonase | pga | K01184 |
|  |  |  | K01213 |
|  | Pectinesterase | pme | K01051 |
| Chitin | Chitin deacetylase | CDA | K01452 |
|  | Chitinase | chi | K01183 |
|  |  |  | K13381 |
|  |  |  | K20547 |
|  | Alpha-N-acetylglucosaminidase | NAGLU | K01205 |

**Table S4 (continued).**

| C source | Enzymes | Abbr. | KO |
| --- | --- | --- | --- |
| Aromatics | Camphor 5-monooxygenase | camDCAB | K19649 |
|  |  |  | K21569 |
|  |  |  | K21570 |
|  |  |  | K26398 |
|  | CDP-diacylglycerol pyrophosphatase | cdh | K01521 |
|  | Vanillate O-demethylase oxygenase | ligXa | K15060 |
|  | limonene-1,2-epoxide hydrolase | limEH | K10533 |
|  | Vanillin dehydrogenase | vdh | K21802 |
| Lignin | Glyoxal oxidase | glx | K20929 |
|  | Lignin peroxidase | lpo | K23515 |
|  | Manganese peroxidase | mnp | K20205 |
|  | Polyphenol oxidase | ppo | K00422 |

Table S5 Fitted parameters for the nonlinear regression analysis between DCF rates and environmental variables including temperature and water content based on generalized additive model (GAM).

|  | | Control treatment | Warming treatment |
| --- | --- | --- | --- |
| Formula | | DCF rates ~ s(Temperature, Water content) | |
| Intercept | coefficient | 0.48 | 0.37 |
|  | SD | 0.03 | 0.02 |
|  | t value | 15.94 | 15.36 |
|  | *P* value | 0.00 | 0.00 |
| Effect level | Df | 6.31 | 5.96 |
|  | *F* | 9.96 | 11.20 |
|  | *P* value | 3.86E-06 | 2.06E-06 |
| Adjust *R*^2^ | | 0.72 | 0.73 |
| Deviance explained | | 0.77 | 0.78 |

Table S6 Climate warming effects on environmental physicochemical parameters in coastal wetland soils based on linear mixed-effects models (LMMs).

| Variable | Ah layer | | | |  | Bh layer | | | |
| --- | --- | --- | --- | --- | --- | --- | --- | --- | --- |
|  | β | *P* | CI (95%) | *R*^2^ |  | β | *P* | CI (95%) | *R*^2^ |
| WC | -0.46 | 0.08 | (-0.97, 0.06) | 0.56 |  | -0.42 | 0.27 | (-0.95, 0.11) | 0.53 |
| EC | -0.29 | 0.42 | (-0.98, 0.41) | 0.00 |  | -0.54 | 0.35 | (-1.21, 0.14) | 0.00 |
| pH | 0.69 | 0.04 | (0.04, 1.35) | 0.36 |  | 0.90 | 0.31 | (0.28, 1.53) | 0.04 |
| NH_4_^+^ | -0.23 | 0.25 | (-0.62, 0.16) | 0.89 |  | 0.25 | 0.23 | (-0.20, 0.70) | 0.76 |
| NO_2_^−^ | 0.47 | 0.03 | (0.03, 0.90) | 0.75 |  | 0.32 | 0.24 | (-0.16, 0.80) | 0.68 |
| NO_3_^−^ | 0.34 | 0.04 | (0.01, 0.66) | 0.99 |  | 0.78 | 0.21 | (0.37, 1.19) | 0.66 |
| TOC | -0.02 | 0.94 | (-0.48, 0.44) | 0.19 |  | 0.11 | 0.23 | (-0.35, 0.57) | 0.17 |
| TN | -0.04 | 0.84 | (-0.45, 0.37) | 0.42 |  | 0.10 | 0.22 | (-0.33, 0.53) | 0.33 |

Note: β denotes the regression coefficients; CI denotes 95% confidence interval of the regression coefficients; *R*^2^ denotes the random effects variance of seasons.

Table S7 Significant tests of the effects of climate warming on the environmental physicochemical parameters in coastal wetland soils.

| Parameter | Group | Z test  *P* value  (Control) | Z test  *P* value  (Warming) | F test  *P* value | t test  *P* value | t test  *P* significance | t test method |
| --- | --- | --- | --- | --- | --- | --- | --- |
| Water content | Spring Ah | 0.658 | 0.909 | 0.093 | 0.155 | ns | Two Sample t-test |
|  | Spring Bh | 0.917 | 0.812 | 0.632 | 0.513 | ns | Two Sample t-test |
|  | Summer Ah | 0.865 | 0.973 | 0.469 | 0.296 | ns | Two Sample t-test |
|  | Summer Bh | 0.767 | 0.924 | 0.183 | 0.028 | * | Two Sample t-test |
|  | Autumn Ah | 0.935 | 0.927 | 0.757 | 0.901 | ns | Two Sample t-test |
|  | Autumn Bh | 0.898 | 0.863 | 0.967 | 0.421 | ns | Two Sample t-test |
|  | Winter Ah | 0.954 | 0.976 | 0.200 | 0.448 | ns | Two Sample t-test |
|  | Winter Bh | 0.974 | 0.805 | 0.009 | 0.906 | ns | Welch Two Sample t-test |
| EC | Spring Ah | 0.686 | 0.182 | 0.864 | 0.093 | ns | Two Sample t-test |
|  | Spring Bh | 0.776 | 0.394 | 0.841 | 0.603 | ns | Two Sample t-test |
|  | Summer Ah | 0.200 | 0.281 | 0.237 | 0.705 | ns | Two Sample t-test |
|  | Summer Bh | 0.307 | 0.461 | 0.435 | 0.499 | ns | Two Sample t-test |
|  | Autumn Ah | 0.491 | 0.522 | 0.687 | 0.646 | ns | Two Sample t-test |
|  | Autumn Bh | 0.328 | 0.822 | 0.920 | 0.674 | ns | Two Sample t-test |
|  | Winter Ah | 0.701 | 0.658 | 0.316 | 0.854 | ns | Two Sample t-test |
|  | Winter Bh | 0.708 | 0.710 | 0.801 | 0.551 | ns | Two Sample t-test |
| pH | Spring Ah | 0.997 | 0.692 | 0.800 | 0.479 | ns | Two Sample t-test |
|  | Spring Bh | 0.275 | 0.462 | 0.797 | 0.622 | ns | Two Sample t-test |
|  | Summer Ah | 0.483 | 0.351 | 0.309 | 0.221 | ns | Two Sample t-test |
|  | Summer Bh | 0.034 | 0.628 | 0.939 | 0.029 | * | Mann-Whitney test |
|  | Autumn Ah | 0.689 | 0.547 | 0.413 | 0.655 | ns | Two Sample t-test |
|  | Autumn Bh | 0.937 | 0.700 | 0.697 | 0.516 | ns | Two Sample t-test |
|  | Winter Ah | 0.405 | 0.923 | 0.700 | 0.532 | ns | Two Sample t-test |
|  | Winter Bh | 0.445 | 0.726 | 0.777 | 0.529 | ns | Two Sample t-test |
| NH_4_^+^ | Spring Ah | 0.203 | 0.702 | 0.935 | 0.161 | ns | Two Sample t-test |
|  | Spring Bh | 0.244 | 0.006 | 0.168 | 0.057 | ns | Mann-Whitney test |
|  | Summer Ah | 0.713 | 0.214 | 0.260 | 0.460 | ns | Two Sample t-test |
|  | Summer Bh | 0.209 | 0.292 | 0.587 | 0.329 | ns | Two Sample t-test |
|  | Autumn Ah | 0.888 | 0.119 | 0.623 | 0.440 | ns | Two Sample t-test |
|  | Autumn Bh | 0.300 | 0.774 | 0.246 | 0.121 | ns | Two Sample t-test |
|  | Winter Ah | 0.330 | 0.459 | 0.003 | 0.035 | * | Welch Two Sample t-test |
|  | Winter Bh | 0.010 | 0.290 | 0.688 | 0.200 | ns | Mann-Whitney test |

**Table S7 (continued).**

| Parameter | Group | Z test  *P* value  (Control) | Z test  *P* value  (Warming) | F test  *P* value | t test  *P* value | t test  *P* significance | t test method |
| --- | --- | --- | --- | --- | --- | --- | --- |
| NO_2_^−^ | Spring Ah | 0.020 | 0.645 | 0.828 | 0.886 | ns | Mann-Whitney test |
|  | Spring Bh | 0.676 | 0.363 | 0.233 | 0.668 | ns | Two Sample t-test |
|  | Summer Ah | 0.198 | 0.406 | 0.948 | 0.086 | ns | Two Sample t-test |
|  | Summer Bh | 0.155 | 0.137 | 0.029 | 0.288 | ns | Welch Two Sample t-test |
|  | Autumn Ah | 0.396 | 0.800 | 0.441 | 0.047 | * | Two Sample t-test |
|  | Autumn Bh | 0.957 | 0.486 | 0.783 | 0.146 | ns | Two Sample t-test |
|  | Winter Ah | 0.898 | 0.425 | 0.015 | 0.826 | ns | Welch Two Sample t-test |
|  | Winter Bh | 0.929 | 0.879 | 0.093 | 0.808 | ns | Two Sample t-test |
| NO_3_^−^ | Spring Ah | 0.563 | 0.614 | 0.458 | 0.483 | ns | Two Sample t-test |
|  | Spring Bh | 0.035 | 0.008 | 0.017 | 0.343 | ns | Mann-Whitney test |
|  | Summer Ah | 0.084 | 0.709 | 0.057 | 0.057 | ns | Mann-Whitney test |
|  | Summer Bh | 0.174 | 0.982 | 0.000 | 0.043 | * | Welch Two Sample t-test |
|  | Autumn Ah | 0.491 | 0.121 | 0.082 | 0.245 | ns | Two Sample t-test |
|  | Autumn Bh | 0.625 | 0.450 | 0.024 | 0.029 | * | Welch Two Sample t-test |
|  | Winter Ah | 0.012 | 0.250 | 0.040 | 0.343 | ns | Mann-Whitney test |
|  | Winter Bh | 0.308 | 0.225 | 0.012 | 0.014 | * | Welch Two Sample t-test |
| TOC | Spring Ah | 0.856 | 0.169 | 0.412 | 0.684 | ns | Two Sample t-test |
|  | Spring Bh | 0.937 | 0.542 | 0.140 | 0.050 | * | Two Sample t-test |
|  | Summer Ah | 0.633 | 0.860 | 0.602 | 0.407 | ns | Two Sample t-test |
|  | Summer Bh | 0.406 | 0.040 | 0.317 | 0.089 | ns | Mann-Whitney test |
|  | Autumn Ah | 0.594 | 0.789 | 0.350 | 0.029 | * | Two Sample t-test |
|  | Autumn Bh | 0.403 | 0.762 | 0.929 | 0.037 | * | Two Sample t-test |
|  | Winter Ah | 0.336 | 0.289 | 0.621 | 0.000 | *** | Two Sample t-test |
|  | Winter Bh | 0.548 | 0.154 | 0.035 | 0.213 | ns | Welch Two Sample t-test |
| TN | Spring Ah | 0.385 | 0.048 | 0.013 | 0.798 | ns | Mann-Whitney test |
|  | Spring Bh | 0.017 | 0.804 | 0.634 | 0.050 | * | Mann-Whitney test |
|  | Summer Ah | 0.627 | 0.085 | 0.012 | 0.141 | ns | Mann-Whitney test |
|  | Summer Bh | 0.141 | 0.078 | 0.338 | 0.204 | ns | Mann-Whitney test |
|  | Autumn Ah | 0.695 | 0.704 | 0.373 | 0.034 | * | Two Sample t-test |
|  | Autumn Bh | 0.209 | 0.154 | 0.388 | 0.915 | ns | Two Sample t-test |
|  | Winter Ah | 0.980 | 0.263 | 0.484 | 0.753 | ns | Two Sample t-test |
|  | Winter Bh | 0.784 | 0.530 | 0.086 | 0.103 | ns | Two Sample t-test |

Note: Significance levels are denoted by asterisks (**P* < 0.05, ***P* < 0.01, and ****P* < 0.001).

Table S8 The relationship between environmental variables and microbial community compositions based on PERMANOVA analyses with 999 permutations.

| Variable | Spring | |  | Summer | |  | Autumn | |  | Winter | |
| --- | --- | --- | --- | --- | --- | --- | --- | --- | --- | --- | --- |
|  | F | *P* |  | F | *P* |  | F | *P* |  | F | *P* |
| Warming | 3.40 | 0.001 |  | 3.25 | 0.001 |  | 4.88 | 0.001 |  | 3.10 | 0.003 |
| Layer | 4.38 | 0.001 |  | 1.75 | 0.001 |  | 6.00 | 0.001 |  | 1.24 | 0.133 |
| WC | 10.21 | 0.001 |  | 2.06 | 0.001 |  | 1.76 | 0.028 |  | 0.99 | 0.394 |
| EC | 3.08 | 0.001 |  | 1.25 | 0.023 |  | 3.87 | 0.001 |  | 1.45 | 0.062 |
| pH | 2.26 | 0.012 |  | 2.49 | 0.001 |  | 2.36 | 0.005 |  | 1.71 | 0.024 |
| NH_4_^+^ | 1.04 | 0.324 |  | 1.34 | 0.009 |  | 2.91 | 0.002 |  | 3.84 | 0.001 |
| NO_2_^−^ | 2.40 | 0.006 |  | 1.63 | 0.002 |  | 6.95 | 0.001 |  | 1.38 | 0.072 |
| NO_3_^−^ | 2.08 | 0.014 |  | 2.42 | 0.001 |  | 3.41 | 0.001 |  | 2.29 | 0.002 |
| TOC | 4.37 | 0.001 |  | 1.74 | 0.001 |  | 2.91 | 0.001 |  | 2.12 | 0.010 |
| TN | 2.21 | 0.014 |  | 1.79 | 0.001 |  | 2.82 | 0.001 |  | 1.84 | 0.017 |

Table S9 Climate warming effects on microbial richness and diversity in coastal wetland soils based on linear mixed-effects models (LMMs).

| Microbial taxa | Chao1 in Ah layer | | | |  | Shannon in Ah layer | | | |  | Chao1 in Bh layer | | | |  | Shannon in Bh layer | | | |
| --- | --- | --- | --- | --- | --- | --- | --- | --- | --- | --- | --- | --- | --- | --- | --- | --- | --- | --- | --- |
|  | β | *P* | CI (95%) | *R*^2^ |  | β | *P* | CI (95%) | *R*^2^ |  | β | *P* | CI (95%) | *R*^2^ |  | β | *P* | CI (95%) | *R*^2^ |
| *Alphaproteobacteria* | -0.36 | 0.00 | (-0.55, -0.16) | 0.94 |  | -0.51 | 0.00 | (-0.71, -0.30) | 0.86 |  | 0.24 | 0.07 | (-0.02, 0.49) | 0.71 |  | 0.27 | 0.06 | (-0.01, 0.55) | 0.49 |
| *Gammaproteobacteria* | -0.01 | 0.90 | (-0.16, 0.14) | 1.14 |  | -0.16 | 0.03 | (-0.31, -0.01) | 1.12 |  | 0.17 | 0.01 | (0.04, 0.29) | 1.17 |  | 0.37 | 0.00 | (0.22, 0.53) | 1.03 |
| *Acidobacteriae* | 0.07 | 0.41 | (-0.09, 0.23) | 1.11 |  | 0.28 | 0.00 | (0.12, 0.44) | 1.09 |  | 0.34 | 0.00 | (0.19, 0.49) | 1.10 |  | 0.42 | 0.00 | (0.26, 0.58) | 1.05 |
| *Blastocatellia* | 0.14 | 0.34 | (-0.15, 0.44) | 0.60 |  | 0.07 | 0.62 | (-0.22, 0.37) | 0.62 |  | 0.61 | 0.00 | (0.34, 0.89) | 0.58 |  | 0.57 | 0.00 | (0.30, 0.84) | 0.58 |
| *Subgroup_22* | 0.10 | 0.17 | (-0.04, 0.24) | 1.15 |  | 0.05 | 0.50 | (-0.10, 0.20) | 1.15 |  | 0.08 | 0.26 | (-0.06, 0.23) | 1.14 |  | 0.10 | 0.12 | (-0.03, 0.22) | 1.19 |
| *Thermoanaerobaculia* | 0.02 | 0.82 | (-0.17, 0.22) | 1.01 |  | 0.43 | 0.00 | (0.24, 0.61) | 0.98 |  | 0.08 | 0.44 | (-0.12, 0.28) | 0.98 |  | 0.13 | 0.17 | (-0.06, 0.31) | 1.02 |
| *Vicinamibacteria* | 0.18 | 0.15 | (-0.07, 0.44) | 0.79 |  | 0.11 | 0.44 | (-0.17, 0.40) | 0.66 |  | 0.28 | 0.01 | (0.06, 0.50) | 0.89 |  | 0.37 | 0.00 | (0.15, 0.60) | 0.78 |
| *Anaerolineae* | -0.06 | 0.57 | (-0.25, 0.14) | 1.00 |  | -0.27 | 0.03 | (-0.51, -0.03) | 0.81 |  | 0.02 | 0.77 | (-0.14, 0.19) | 1.11 |  | 0.20 | 0.09 | (-0.03, 0.42) | 0.85 |
| *Chloroflexia* | -0.15 | 0.18 | (-0.38, 0.07) | 0.89 |  | -0.24 | 0.01 | (-0.44, -0.05) | 1.00 |  | 0.27 | 0.02 | (0.05, 0.49) | 0.91 |  | 0.09 | 0.36 | (-0.10, 0.27) | 1.04 |
| *Dehalococcoidia* | 0.20 | 0.08 | (-0.03, 0.42) | 0.91 |  | -0.16 | 0.20 | (-0.42, 0.09) | 0.79 |  | -0.02 | 0.84 | (-0.24, 0.20) | 0.93 |  | -0.04 | 0.81 | (-0.33, 0.26) | 0.62 |
| *JG30-KF-CM66* | 0.26 | 0.03 | (0.02, 0.51) | 0.82 |  | 0.02 | 0.86 | (-0.22, 0.26) | 0.86 |  | 0.09 | 0.39 | (-0.11, 0.29) | 0.98 |  | 0.00 | 0.98 | (-0.20, 0.21) | 0.98 |
| *KD4-96* | 0.14 | 0.24 | (-0.10, 0.39) | 0.81 |  | -0.20 | 0.09 | (-0.43, 0.03) | 0.87 |  | 0.09 | 0.38 | (-0.11, 0.29) | 1.00 |  | -0.05 | 0.70 | (-0.31, 0.21) | 0.76 |
| *Acidimicrobiia* | -0.42 | 0.00 | (-0.60, -0.24) | 1.00 |  | -0.48 | 0.00 | (-0.69, -0.27) | 0.88 |  | 0.01 | 0.93 | (-0.18, 0.19) | 1.04 |  | 0.06 | 0.52 | (-0.13, 0.26) | 0.99 |
| *Actinobacteria* | -0.13 | 0.17 | (-0.33, 0.06) | 1.02 |  | -0.36 | 0.00 | (-0.56, -0.15) | 0.94 |  | 0.34 | 0.00 | (0.15, 0.52) | 1.01 |  | 0.08 | 0.46 | (-0.14, 0.30) | 0.92 |
| *MB-A2-108* | -0.08 | 0.55 | (-0.35, 0.19) | 0.59 |  | 0.04 | 0.80 | (-0.25, 0.33) | 0.55 |  | 0.32 | 0.02 | (0.05, 0.60) | 0.64 |  | 0.28 | 0.06 | (-0.01, 0.58) | 0.57 |
| *Thermoleophilia* | -0.01 | 0.90 | (-0.21, 0.19) | 0.98 |  | 0.09 | 0.31 | (-0.09, 0.27) | 1.04 |  | 0.20 | 0.07 | (-0.02, 0.43) | 0.86 |  | 0.28 | 0.01 | (0.05, 0.50) | 0.89 |
| *OM190* | 0.23 | 0.04 | (0.00, 0.46) | 0.89 |  | 0.25 | 0.03 | (0.03, 0.47) | 0.90 |  | 0.30 | 0.01 | (0.07, 0.53) | 0.80 |  | 0.21 | 0.08 | (-0.02, 0.43) | 0.82 |
| *Phycisphaerae* | -0.08 | 0.46 | (-0.31, 0.14) | 0.91 |  | -0.11 | 0.32 | (-0.33, 0.11) | 0.92 |  | 0.31 | 0.01 | (0.07, 0.55) | 0.81 |  | 0.37 | 0.00 | (0.15, 0.58) | 0.86 |
| *Planctomycetes* | -0.10 | 0.36 | (-0.32, 0.12) | 0.93 |  | -0.16 | 0.07 | (-0.33, 0.01) | 1.07 |  | 0.18 | 0.06 | (-0.01, 0.37) | 0.97 |  | 0.20 | 0.02 | (0.03, 0.37) | 1.03 |
| *Bacilli* | -0.28 | 0.02 | (-0.51, -0.05) | 0.87 |  | -0.13 | 0.27 | (-0.36, 0.10) | 0.87 |  | -0.18 | 0.20 | (-0.46, 0.10) | 0.66 |  | 0.10 | 0.28 | (-0.09, 0.30) | 1.00 |
| *Clostridia* | -0.55 | 0.00 | (-0.84, -0.26) | 0.52 |  | -0.41 | 0.02 | (-0.75, -0.07) | 0.33 |  | -0.61 | 0.00 | (-0.87, -0.35) | 0.65 |  | -0.58 | 0.00 | (-0.86, -0.30) | 0.56 |
| *Desulfobacterota* | -0.05 | 0.53 | (-0.22, 0.11) | 1.10 |  | -0.90 | 0.00 | (-1.11, -0.69) | 0.70 |  | -0.21 | 0.02 | (-0.39, -0.04) | 1.05 |  | 0.02 | 0.94 | (-0.36, 0.39) | 0.17 |
| *Bacteroidota* | -0.13 | 0.10 | (-0.28, 0.03) | 1.10 |  | -0.04 | 0.55 | (-0.19, 0.10) | 1.12 |  | 0.05 | 0.63 | (-0.15, 0.24) | 0.97 |  | 0.13 | 0.21 | (-0.07, 0.33) | 0.88 |
| *Myxococcota* | -0.06 | 0.47 | (-0.22, 0.10) | 1.10 |  | -0.23 | 0.00 | (-0.37, -0.09) | 1.15 |  | 0.14 | 0.11 | (-0.03, 0.31) | 1.07 |  | 0.23 | 0.00 | (0.09, 0.37) | 1.14 |
| *Crenarchaeota* | 0.44 | 0.00 | (0.21, 0.66) | 0.81 |  | 0.11 | 0.40 | (-0.15, 0.38) | 0.76 |  | 0.26 | 0.01 | (0.07, 0.44) | 1.01 |  | -0.01 | 0.91 | (-0.21, 0.18) | 0.99 |
| *Gemmatimonadota* | 0.12 | 0.18 | (-0.06, 0.30) | 1.06 |  | -0.12 | 0.29 | (-0.33, 0.10) | 0.94 |  | 0.26 | 0.01 | (0.08, 0.44) | 1.02 |  | 0.31 | 0.00 | (0.10, 0.51) | 0.94 |
| *NB1-j* | -0.09 | 0.23 | (-0.23, 0.06) | 1.12 |  | -0.27 | 0.00 | (-0.41, -0.13) | 1.10 |  | 0.05 | 0.48 | (-0.09, 0.20) | 1.15 |  | 0.05 | 0.58 | (-0.12, 0.21) | 1.07 |
| *Verrucomicrobiota* | 0.05 | 0.69 | (-0.18, 0.27) | 0.91 |  | -0.40 | 0.00 | (-0.57, -0.22) | 1.01 |  | 0.21 | 0.04 | (0.01, 0.41) | 0.91 |  | 0.09 | 0.34 | (-0.10, 0.28) | 0.97 |
| *MBNT15* | -0.15 | 0.05 | (-0.31, 0.00) | 1.10 |  | -0.32 | 0.00 | (-0.52, -0.12) | 0.97 |  | -0.29 | 0.00 | (-0.47, -0.12) | 1.05 |  | 0.22 | 0.03 | (0.02, 0.43) | 0.93 |
| *Latescibacterota* | 0.19 | 0.05 | (0.00, 0.39) | 1.01 |  | 0.01 | 0.95 | (-0.16, 0.18) | 1.08 |  | 0.12 | 0.12 | (-0.03, 0.28) | 1.12 |  | 0.24 | 0.01 | (0.07, 0.41) | 1.06 |
| *Methylomirabilota* | 0.13 | 0.29 | (-0.12, 0.38) | 0.68 |  | -0.01 | 0.96 | (-0.30, 0.28) | 0.40 |  | 0.34 | 0.01 | (0.07, 0.61) | 0.62 |  | 0.45 | 0.01 | (0.13, 0.77) | 0.31 |
| *Nitrospirota* | 0.30 | 0.00 | (0.10, 0.50) | 0.95 |  | 0.31 | 0.00 | (0.13, 0.48) | 1.02 |  | -0.11 | 0.28 | (-0.30, 0.09) | 1.02 |  | 0.03 | 0.74 | (-0.15, 0.21) | 1.03 |
| Total | -0.09 | 0.17 | (-0.23, 0.04) | 1.17 |  | -0.21 | 0.01 | (-0.37, -0.04) | 1.08 |  | 0.16 | 0.02 | (0.02, 0.29) | 1.15 |  | 0.22 | 0.01 | (0.06, 0.38) | 1.04 |

Note: β denotes the regression coefficients; CI denotes 95% confidence interval of the regression coefficients; *R*^2^ denotes the random effects variance of seasons.

Table S10 Significant tests of the effects of climate warming on the dark carbon fixation (DCF) rates in coastal wetland soils.

| Group | Z test  *P* value  (Control) | Z test  *P* value  (Warming) | F test  *P* value | t test  *P* value | t test  *P* significance | t test method |
| --- | --- | --- | --- | --- | --- | --- |
| Spring Ah | 0.033 | 0.926 | 0.327 | 0.093 | ns | Mann-Whitney test |
| Spring Bh | 0.691 | 0.485 | 0.880 | 0.578 | ns | Two Sample t-test |
| Summer Ah | 0.633 | 0.837 | 0.808 | 0.085 | ns | Two Sample t-test |
| Summer Bh | 0.506 | 0.080 | 0.073 | 0.008 | ** | Mann-Whitney test |
| Autumn Ah | 0.342 | 0.602 | 0.085 | 0.427 | ns | Two Sample t-test |
| Autumn Bh | 0.462 | 0.501 | 0.450 | 0.503 | ns | Two Sample t-test |
| Winter Ah | 0.178 | 0.290 | 0.011 | 0.000 | *** | Welch Two Sample t-test |
| Winter Bh | 0.041 | 0.057 | 0.010 | 0.002 | ** | Mann-Whitney test |

Note: Significance levels are denoted by asterisks (**P* < 0.05, ***P* < 0.01, and ****P* < 0.001).

Table S11 Significant tests of the effects of climate warming on abundance of chemoautotrophic functional genes in coastal wetland soils.

| Gene | Group | Z test  *P* value  (Control) | Z test  *P* value  (Warming) | F test  *P* value | t test  *P* value | t test  *P* significance | t test method |
| --- | --- | --- | --- | --- | --- | --- | --- |
| *cbbL* | Spring Ah | 0.183 | 0.105 | 0.564 | 0.018 | * | Two Sample t-test |
|  | Spring Bh | 0.674 | 0.676 | 0.024 | 0.127 | ns | Welch Two Sample t-test |
|  | Summer Ah | 0.004 | 0.817 | 0.261 | 0.234 | ns | Mann-Whitney test |
|  | Summer Bh | 0.586 | 0.891 | 0.456 | 0.001 | *** | Two Sample t-test |
|  | Autumn Ah | 0.380 | 0.700 | 0.186 | 0.004 | ** | Two Sample t-test |
|  | Autumn Bh | 0.014 | 0.421 | 0.950 | 0.574 | ns | Mann-Whitney test |
|  | Winter Ah | 0.005 | 0.859 | 0.622 | 1.000 | ns | Mann-Whitney test |
|  | Winter Bh | 0.106 | 0.120 | 0.065 | 0.797 | ns | Two Sample t-test |
| *cbbM* | Spring Ah | 0.223 | 0.671 | 0.016 | 0.002 | ** | Welch Two Sample t-test |
|  | Spring Bh | 0.018 | 0.142 | 0.913 | 0.442 | ns | Mann-Whitney test |
|  | Summer Ah | 0.862 | 0.092 | 0.066 | 0.231 | ns | Two Sample t-test |
|  | Summer Bh | 0.174 | 0.003 | 0.049 | 0.161 | ns | Mann-Whitney test |
|  | Autumn Ah | 0.705 | 0.853 | 0.999 | 0.023 | * | Two Sample t-test |
|  | Autumn Bh | 0.255 | 0.306 | 0.223 | 0.075 | ns | Two Sample t-test |
|  | Winter Ah | 0.258 | 0.641 | 0.514 | 0.284 | ns | Two Sample t-test |
|  | Winter Bh | 0.068 | 0.138 | 0.210 | 0.327 | ns | Two Sample t-test |
| *aclB* | Spring Ah | 0.469 | 0.990 | 0.010 | 0.026 | * | Welch Two Sample t-test |
|  | Spring Bh | 0.189 | 0.811 | 0.023 | 0.117 | ns | Welch Two Sample t-test |
|  | Summer Ah | 0.091 | 0.837 | 0.293 | 0.354 | ns | Two Sample t-test |
|  | Summer Bh | 0.463 | 0.239 | 0.208 | 0.009 | ** | Two Sample t-test |
|  | Autumn Ah | 0.109 | 0.848 | 0.271 | 0.031 | * | Two Sample t-test |
|  | Autumn Bh | 0.177 | 0.452 | 0.232 | 0.066 | ns | Two Sample t-test |
|  | Winter Ah | 0.671 | 0.542 | 0.045 | 0.278 | ns | Welch Two Sample t-test |
|  | Winter Bh | 0.987 | 0.434 | 0.006 | 0.364 | ns | Welch Two Sample t-test |
| *hbd* | Spring Ah | 0.281 | 0.010 | 0.746 | 0.130 | ns | Mann-Whitney test |
|  | Spring Bh | 0.354 | 0.018 | 0.088 | 0.050 | * | Mann-Whitney test |
|  | Summer Ah | 0.327 | 0.950 | 0.111 | 0.006 | ** | Two Sample t-test |
|  | Summer Bh | 0.321 | 0.060 | 0.000 | 0.427 | ns | Welch Two Sample t-test |
|  | Autumn Ah | 0.125 | 0.822 | 0.000 | 0.170 | ns | Welch Two Sample t-test |
|  | Autumn Bh | 0.483 | 0.255 | 0.021 | 0.031 | * | Welch Two Sample t-test |
|  | Winter Ah | 0.001 | 0.491 | 0.944 | 0.021 | * | Mann-Whitney test |
|  | Winter Bh | 0.669 | 0.096 | 0.001 | 0.082 | ns | Welch Two Sample t-test |

**Table S11** **(continued).**

| Gene | Group | Z test  *P* value  (Control) | Z test  *P* value  (Warming) | F test  *P* value | t test  *P* value | t test  *P* significance | t test method |
| --- | --- | --- | --- | --- | --- | --- | --- |
| *accA* | Spring Ah | 0.251 | 0.356 | 0.924 | 0.528 | ns | Two Sample t-test |
|  | Spring Bh | 0.002 | 0.104 | 0.298 | 0.721 | ns | Mann-Whitney test |
|  | Summer Ah | 0.001 | 0.002 | 0.203 | 0.038 | * | Mann-Whitney test |
|  | Summer Bh | 0.094 | 0.546 | 0.160 | 0.141 | ns | Two Sample t-test |
|  | Autumn Ah | 0.076 | 0.537 | 0.001 | 0.074 | ns | Welch Two Sample t-test |
|  | Autumn Bh | 0.142 | 0.310 | 0.007 | 0.017 | * | Welch Two Sample t-test |
|  | Winter Ah | 0.002 | 0.606 | 0.784 | 0.574 | ns | Mann-Whitney test |
|  | Winter Bh | 0.711 | 0.307 | 0.000 | 0.286 | ns | Welch Two Sample t-test |

Note: Significance levels are denoted by asterisks (**P* < 0.05, ***P* < 0.01, and ****P* < 0.001).

Table S12 Climate warming effects on the normalized abundance of major chemoautotrophic lineages in coastal wetland soils based on linear mixed-effects models (LMMs).

| Chemoautotrophs | Ah layer | | | |  | Bh layer | | | |
| --- | --- | --- | --- | --- | --- | --- | --- | --- | --- |
|  | β | *P* | CI (95%) | *R*^2^ |  | β | *P* | CI (95%) | *R*^2^ |
| *Gammaproteobacteria* | -0.78 | 0.01 | (-1.36, -0.20) | 0.24 |  | -0.68 | 0.34 | (-1.34, -0.02) | 0.00 |
| *Alphaproteobacteria* | -0.91 | 0.00 | (-1.47, -0.35) | 0.23 |  | -0.59 | 0.28 | (-1.15, -0.03) | 0.39 |
| *Actinobacteria* | -0.48 | 0.12 | (-1.09, 0.13) | 0.28 |  | 0.09 | 0.31 | (-0.53, 0.72) | 0.30 |
| *Deltaproteobacteria* | -0.48 | 0.11 | (-1.09, 0.12) | 0.30 |  | 0.00 | 0.32 | (-0.63, 0.63) | 0.29 |
| *Thaumarchaeota* | 0.77 | 0.01 | (0.16, 1.37) | 0.17 |  | 0.30 | 0.34 | (-0.39, 0.99) | 0.07 |
| *Nitrospirae* | 0.61 | 0.04 | (0.01, 1.21) | 0.27 |  | 0.41 | 0.35 | (-0.28, 1.10) | 0.00 |

Note: β denotes the regression coefficients; CI denotes 95% confidence interval of the regression coefficients; *R*^2^ denotes the random effects variance of seasons.

Table S13 Climate warming effects on various carbon fixation pathways in coastal wetland soils based on linear mixed-effects models (LMMs).

| Pathway | Ah layer | | | |  | Bh layer | | | |
| --- | --- | --- | --- | --- | --- | --- | --- | --- | --- |
|  | β | *P* | CI (95%) | *R*^2^ |  | β | *P* | CI (95%) | *R*^2^ |
| CBB | -0.92 | 0.00 | (-1.50, -0.35) | 0.18 |  | -0.90 | 0.29 | (-1.48, -0.31) | 0.17 |
| W-L | -0.65 | 0.05 | (-1.30, 0.00) | 0.08 |  | -0.42 | 0.32 | (-1.05, 0.21) | 0.23 |
| rTCA | 0.03 | 0.92 | (-0.49, 0.54) | 0.63 |  | 0.05 | 0.28 | (-0.51, 0.61) | 0.51 |
| 3HP | -0.58 | 0.09 | (-1.25, 0.10) | 0.00 |  | -0.15 | 0.34 | (-0.84, 0.54) | 0.10 |
| 3HP/4HB | 0.77 | 0.01 | (0.16, 1.37) | 0.17 |  | 0.30 | 0.34 | (-0.39, 0.99) | 0.07 |
| DC/4HB | 0.02 | 0.95 | (-0.68, 0.73) | 0.04 |  | -0.08 | 0.33 | (-0.74, 0.58) | 0.19 |

Note: β denotes the regression coefficients; CI denotes 95% confidence interval of the regression coefficients; *R*^2^ denotes the random effects variance of seasons.

Table S14 Climate warming effects on the richness (Chao1), diversity (Shannon), and evenness (Pielou) of chemoautotrophic communities using different carbon fixation pathways in coastal wetland soils based on linear mixed-effects models (LMMs).

| Pathway | Indices | Ah layer | | | |  | Bh layer | | | |
| --- | --- | --- | --- | --- | --- | --- | --- | --- | --- | --- |
|  |  | β | *P* | CI (95%) | *R*^2^ |  | β | *P* | CI (95%) | *R*^2^ |
| CBB | Chao1 | -0.82 | 0.00 | (-1.30, -0.34) | 0.49 |  | -0.88 | 0.00 | (-1.45, -0.32) | 0.23 |
|  | Shannon | -0.93 | 0.00 | (-1.40, -0.46) | 0.44 |  | -0.92 | 0.00 | (-1.52, -0.33) | 0.10 |
|  | Pielou | 0.02 | 0.91 | (-0.42, 0.47) | 0.80 |  | 0.12 | 0.57 | (-0.29, 0.52) | 0.87 |
| W-L | Chao1 | -0.66 | 0.04 | (-1.30, -0.01) | 0.10 |  | -0.41 | 0.15 | (-0.97, 0.15) | 0.44 |
|  | Shannon | -0.55 | 0.10 | (-1.21, 0.11) | 0.09 |  | -0.28 | 0.34 | (-0.85, 0.30) | 0.44 |
|  | Pielou | 0.54 | 0.08 | (-0.08, 1.16) | 0.22 |  | 0.20 | 0.43 | (-0.31, 0.72) | 0.61 |
| rTCA | Chao1 | -0.32 | 0.21 | (-0.83, 0.19) | 0.60 |  | -0.57 | 0.04 | (-1.13, -0.01) | 0.40 |
|  | Shannon | -0.24 | 0.32 | (-0.73, 0.24) | 0.68 |  | -0.60 | 0.01 | (-1.08, -0.12) | 0.59 |
|  | Pielou | -0.17 | 0.52 | (-0.68, 0.35) | 0.61 |  | -0.25 | 0.27 | (-0.71, 0.20) | 0.74 |
| 3HP | Chao1 | -0.77 | 0.02 | (-1.42, -0.12) | 0.00 |  | -0.88 | 0.00 | (-1.48, -0.28) | 0.14 |
|  | Shannon | -1.04 | 0.00 | (-1.63, -0.44) | 0.05 |  | -0.42 | 0.16 | (-1.03, 0.18) | 0.31 |
|  | Pielou | -0.35 | 0.25 | (-0.95, 0.25) | 0.35 |  | -0.21 | 0.42 | (-0.72, 0.30) | 0.62 |
| 3HP/4HB | Chao1 | 0.06 | 0.85 | (-0.60, 0.73) | 0.16 |  | 0.27 | 0.45 | (-0.43, 0.97) | 0.00 |
|  | Shannon | 0.28 | 0.43 | (-0.41, 0.98) | 0.00 |  | 0.33 | 0.31 | (-0.32, 0.98) | 0.18 |
|  | Pielou | -0.29 | 0.34 | (-0.91, 0.32) | 0.31 |  | 0.11 | 0.75 | (-0.59, 0.81) | 0.00 |
| DC/4HB | Chao1 | 0.01 | 0.98 | (-0.69, 0.71) | 0.00 |  | -0.05 | 0.89 | (-0.73, 0.64) | 0.10 |
|  | Shannon | 0.13 | 0.72 | (-0.57, 0.82) | 0.06 |  | -0.08 | 0.82 | (-0.73, 0.58) | 0.22 |
|  | Pielou | -0.04 | 0.93 | (-0.94, 0.86) | 0.00 |  | 0.42 | 0.27 | (-0.49, 1.14) | 0.33 |

Note: β denotes the regression coefficients; CI denotes 95% confidence interval of the regression coefficients; *R*^2^ denotes the random effects variance of seasons.

Table S15 Climate warming effects on the normalized abundances of enzymes associated with different carbon fixation pathways in coastal wetland soils based on linear mixed-effects models (LMMs).

| Pathway | Enzyme | Ah layer | | | |  | Bh layer | | | |
| --- | --- | --- | --- | --- | --- | --- | --- | --- | --- | --- |
|  |  | β | *P* | CI (95%) | *R*^2^ |  | β | *P* | CI (95%) | *R*^2^ |
| CBB | RuBisCO | -0.99 | 0.00 | (-1.55, -0.43) | 0.17 |  | -0.88 | 0.00 | (-1.47, -0.29) | 0.15 |
|  | PGK | -0.80 | 0.01 | (-1.39, -0.21) | 0.21 |  | -0.35 | 0.23 | (-0.92, 0.23) | 0.42 |
|  | FBP | -0.57 | 0.09 | (-1.24, 0.10) | 0.06 |  | -0.24 | 0.44 | (-0.85, 0.37) | 0.34 |
|  | SBP | -0.70 | 0.01 | (-1.26, -0.15) | 0.35 |  | -0.33 | 0.18 | (-0.82, 0.16) | 0.65 |
|  | PRK | -1.10 | 0.00 | (-1.62, -0.58) | 0.21 |  | -1.01 | 0.00 | (-1.55, -0.46) | 0.21 |
| W-L | ACS | -0.66 | 0.05 | (-1.32, 0.00) | 0.05 |  | -0.46 | 0.15 | (-1.09, 0.17) | 0.21 |
|  | CODH | -0.78 | 0.01 | (-1.38, -0.18) | 0.19 |  | -0.59 | 0.04 | (-1.15, -0.03) | 0.38 |
|  | FTHFS | -0.47 | 0.05 | (-0.95, 0.00) | 0.65 |  | -0.11 | 0.68 | (-0.64, 0.42) | 0.59 |
|  | AcsE | -0.75 | 0.02 | (-1.40, -0.10) | 0.00 |  | -0.40 | 0.24 | (-1.06, 0.27) | 0.12 |
| rTCA | OOR | -0.50 | 0.11 | (-1.12, 0.12) | 0.23 |  | -0.18 | 0.50 | (-0.71, 0.35) | 0.57 |
|  | Icd | -0.91 | 0.00 | (-1.53, -0.28) | 0.00 |  | -0.22 | 0.53 | (-0.89, 0.46) | 0.12 |
|  | ACL | 0.43 | 0.13 | (-0.13, 1.00) | 0.43 |  | 0.29 | 0.36 | (-0.34, 0.92) | 0.26 |
|  | CCS | -0.16 | 0.57 | (-0.71, 0.40) | 0.51 |  | 0.08 | 0.78 | (-0.49, 0.64) | 0.49 |
|  | MDH | -0.67 | 0.04 | (-1.32, -0.02) | 0.08 |  | -0.26 | 0.38 | (-0.85, 0.33) | 0.39 |
|  | A-SCS | -0.64 | 0.03 | (-1.23, -0.04) | 0.27 |  | -0.23 | 0.41 | (-0.80, 0.33) | 0.48 |
| 3HP | MCR | -0.60 | 0.01 | (-1.06, -0.14) | 0.64 |  | -0.43 | 0.07 | (-0.90, 0.04) | 0.67 |
|  | HPS | -0.68 | 0.00 | (-1.16, -0.20) | 0.56 |  | -0.47 | 0.02 | (-0.89, -0.06) | 0.78 |
|  | MCE | -0.33 | 0.30 | (-0.97, 0.31) | 0.22 |  | 0.04 | 0.88 | (-0.48, 0.56) | 0.62 |
|  | Mcl | -1.15 | 0.00 | (-1.71, -0.59) | 0.05 |  | -0.28 | 0.31 | (-0.83, 0.27) | 0.50 |
|  | Mct | -0.58 | 0.09 | (-1.25, 0.10) | 0.00 |  | -0.15 | 0.66 | (-0.84, 0.54) | 0.10 |
|  | Meh | -0.34 | 0.18 | (-0.84, 0.16) | 0.62 |  | -0.37 | 0.14 | (-0.88, 0.13) | 0.60 |
| 3HP/4HB | ACC | 0.81 | 0.00 | (0.24, 1.39) | 0.24 |  | 0.65 | 0.01 | (0.12, 1.17) | 0.46 |
|  | MCE | 0.41 | 0.20 | (-0.24, 1.06) | 0.18 |  | -0.24 | 0.48 | (-0.90, 0.43) | 0.15 |
|  | MCM | 1.01 | 0.00 | (0.40, 1.61) | 0.03 |  | 0.50 | 0.12 | (-0.14, 1.15) | 0.15 |
|  | HBD | 0.77 | 0.01 | (0.16, 1.37) | 0.17 |  | 0.30 | 0.39 | (-0.39, 0.99) | 0.07 |
|  | HAD | 0.38 | 0.28 | (-0.31, 1.07) | 0.00 |  | 0.14 | 0.66 | (-0.49, 0.76) | 0.30 |
|  | ACAT | 0.77 | 0.01 | (0.15, 1.38) | 0.14 |  | 0.32 | 0.35 | (-0.37, 1.00) | 0.08 |
| DC/4HB | ACAT | 0.02 | 0.95 | (-0.68, 0.73) | 0.04 |  | -0.08 | 0.81 | (-0.74, 0.58) | 0.19 |

Note: β denotes the regression coefficients; CI denotes 95% confidence interval of the regression coefficients; *R*^2^ denotes the random effects variance of seasons.

Table S16 Correlations between environmental parameters and DCF rates, carbon mineralization (CM) rates, abundances and diversity of chemoautotrophic microbial communities, carbon fixation pathways, and majority lineages of chemoautotrophs.

| Variable | Warming | | WC | | EC | | pH | | TOC | | TN | | NH_4_^+^ | | NO_2_^-^ | | NO_3_^-^ | |
| --- | --- | --- | --- | --- | --- | --- | --- | --- | --- | --- | --- | --- | --- | --- | --- | --- | --- | --- |
|  | *r* | *P* | *r* | *P* | *r* | *P* | *r* | *P* | *r* | *P* | *r* | *P* | *r* | *P* | *r* | *P* | *r* | *P* |
| DCF rates | -0.15 | 0.04 | 0.31 | 0.00 | 0.03 | 0.82 | -0.19 | 0.01 | 0.33 | 0.00 | 0.26 | 0.00 | 0.21 | 0.14 | -0.06 | 0.75 | -0.08 | 0.64 |
| CM rates | -0.11 | 0 | 0.19 | 0.00 | 0.03 | 0.64 | -0.11 | 0.01 | 0.12 | 0.01 | 0.08 | 0.19 | 0.01 | 0.93 | -0.02 | 0.83 | -0.09 | 0.32 |
| *cbbL* gene | -0.2 | 0.1 | 0.14 | 0.51 | 0.20 | 0.10 | -0.23 | 0.07 | 0.02 | 0.92 | 0.08 | 0.76 | 0.15 | 0.58 | -0.03 | 0.90 | -0.12 | 0.64 |
| *cbbM* gene | -0.12 | 0.51 | -0.26 | 0.15 | 0.19 | 0.14 | -0.15 | 0.35 | -0.10 | 0.59 | 0.00 | 0.99 | -0.01 | 0.98 | 0.08 | 0.77 | -0.13 | 0.62 |
| *aclB* gene | -0.17 | 0.07 | 0.05 | 0.80 | 0.08 | 0.55 | -0.20 | 0.05 | -0.04 | 0.80 | -0.04 | 0.85 | 0.09 | 0.69 | -0.03 | 0.86 | -0.12 | 0.55 |
| *hbd* gene | -0.13 | 0.44 | 0.18 | 0.36 | 0.13 | 0.45 | -0.05 | 0.83 | -0.11 | 0.58 | -0.15 | 0.47 | -0.03 | 0.91 | 0.02 | 0.93 | -0.04 | 0.89 |
| *accA* gene | -0.08 | 0.67 | -0.09 | 0.70 | 0.18 | 0.17 | -0.42 | 0.00 | -0.11 | 0.58 | 0.13 | 0.59 | 0.13 | 0.61 | -0.03 | 0.90 | -0.08 | 0.75 |
| Chao1 | -0.41 | 0 | 0.24 | 0.23 | 0.04 | 0.85 | -0.07 | 0.73 | -0.10 | 0.62 | -0.13 | 0.51 | -0.13 | 0.64 | -0.38 | 0.01 | -0.20 | 0.34 |
| Shannon | -0.39 | 0 | 0.30 | 0.11 | -0.03 | 0.86 | -0.13 | 0.51 | -0.06 | 0.80 | -0.19 | 0.34 | 0.05 | 0.87 | -0.38 | 0.01 | -0.33 | 0.05 |
| Pielou | 0.03 | 0.81 | -0.11 | 0.47 | 0.02 | 0.86 | -0.11 | 0.26 | -0.10 | 0.41 | -0.16 | 0.14 | 0.03 | 0.88 | -0.03 | 0.88 | -0.24 | 0.07 |
| CBB cycle | -0.37 | 0 | 0.25 | 0.11 | 0.09 | 0.64 | -0.04 | 0.86 | 0.04 | 0.86 | -0.04 | 0.86 | 0.13 | 0.59 | -0.36 | 0.03 | -0.11 | 0.64 |
| W-L pathway | -0.16 | 0.11 | 0.20 | 0.11 | 0.03 | 0.86 | 0.03 | 0.84 | 0.13 | 0.35 | 0.11 | 0.51 | 0.07 | 0.75 | -0.17 | 0.23 | 0.20 | 0.07 |
| rTCA cycle | 0.02 | 0.91 | 0.21 | 0.11 | -0.16 | 0.10 | 0.17 | 0.11 | 0.10 | 0.51 | 0.02 | 0.91 | 0.10 | 0.64 | 0.10 | 0.62 | 0.22 | 0.20 |
| 3HP bicycle | -0.17 | 0.42 | 0.40 | 0.01 | 0.07 | 0.79 | 0.24 | 0.17 | 0.12 | 0.60 | 0.02 | 0.91 | 0.14 | 0.59 | -0.06 | 0.83 | 0.17 | 0.51 |
| 3HP/4HB cycle | 0.25 | 0.08 | 0.03 | 0.89 | 0.00 | 0.99 | -0.07 | 0.75 | -0.08 | 0.73 | 0.15 | 0.55 | -0.20 | 0.39 | 0.29 | 0.05 | 0.16 | 0.51 |
| DC/4HB cycle | -0.02 | 0.91 | -0.38 | 0.04 | 0.21 | 0.23 | -0.11 | 0.64 | -0.15 | 0.51 | -0.07 | 0.80 | -0.27 | 0.12 | -0.22 | 0.35 | -0.12 | 0.68 |
| *Gammaproteobacteria* | -0.28 | 0.01 | 0.11 | 0.59 | 0.09 | 0.62 | -0.02 | 0.91 | 0.06 | 0.77 | -0.05 | 0.83 | 0.17 | 0.37 | -0.29 | 0.07 | -0.18 | 0.35 |
| *Alphaproteobacteria* | -0.35 | 0 | 0.51 | 0.00 | 0.04 | 0.85 | 0.05 | 0.83 | 0.06 | 0.80 | -0.07 | 0.80 | 0.13 | 0.67 | -0.26 | 0.24 | -0.01 | 0.95 |
| *Actinobacteria* | -0.08 | 0.7 | 0.08 | 0.77 | -0.04 | 0.86 | 0.14 | 0.48 | 0.17 | 0.39 | 0.13 | 0.59 | 0.10 | 0.75 | -0.24 | 0.31 | 0.32 | 0.10 |
| *Deltaproteobacteria* | -0.07 | 0.64 | 0.13 | 0.51 | 0.02 | 0.91 | 0.02 | 0.88 | 0.09 | 0.62 | 0.05 | 0.83 | 0.28 | 0.06 | -0.14 | 0.51 | 0.03 | 0.89 |
| *Thaumarchaeota* | 0.25 | 0.08 | 0.03 | 0.89 | 0.00 | 0.99 | -0.07 | 0.75 | -0.08 | 0.73 | 0.15 | 0.55 | -0.20 | 0.39 | 0.29 | 0.05 | 0.16 | 0.51 |
| *Nitrospirae* | 0.26 | 0.11 | 0.15 | 0.59 | 0.06 | 0.80 | 0.02 | 0.91 | 0.00 | 0.99 | 0.11 | 0.64 | 0.03 | 0.91 | -0.05 | 0.86 | 0.30 | 0.10 |

Table S17 Variance of explanation of latent variables to dependent variables, determined using structural equation modelling (SEM) analysis.

| Latent variable | Dependent variable | Coefficient (*b*) | SD | Z value | *P* value | 95% CI |
| --- | --- | --- | --- | --- | --- | --- |
| WC | Warming | -0.31 | 0.11 | -2.84 | 0.00 | (-0.53, -0.10) |
| EC | Warming | -0.21 | 0.12 | -1.76 | 0.08 | (-0.44, 0.02) |
| pH | WC | -0.21 | 0.11 | -1.87 | 0.06 | (-0.43, 0.01) |
|  | TOC | -0.23 | 0.11 | -1.96 | 0.05 | (-0.45, 0.00) |
|  | NO_2_^−^ | 0.16 | 0.11 | 1.42 | 0.15 | (-0.06, 0.38) |
|  | NO_3_^−^ | 0.31 | 0.11 | 2.91 | 0.00 | (0.10, 0.51) |
| TOC | WC | 0.33 | 0.11 | 3.06 | 0.00 | (0.12, 0.55) |
|  | EC | -0.21 | 0.11 | -1.82 | 0.07 | (-0.43, 0.02) |
| NO_2_^−^ | Warming | 0.25 | 0.11 | 2.18 | 0.03 | (0.03, 0.47) |
|  | TOC | 0.23 | 0.12 | 1.97 | 0.05 | (0.00, 0.45) |
| NO_3_^−^ | Warming | 0.64 | 0.07 | 8.58 | 0.00 | (0.49, 0.79) |
|  | WC | 0.11 | 0.10 | 1.02 | 0.31 | (-0.10, 0.31) |
| Gene abundance | EC | 0.19 | 0.11 | 1.78 | 0.07 | (-0.02, 0.40) |
|  | pH | -0.35 | 0.11 | -3.22 | 0.00 | (-0.57, -0.14) |
|  | TOC | 0.26 | 0.11 | 2.47 | 0.01 | (0.05, 0.47) |
|  | NO_3_^−^ | -0.09 | 0.11 | -0.85 | 0.39 | (-0.31, 0.12) |
| Biodiversity | Warming | -0.49 | 0.09 | -5.56 | 0.00 | (-0.67, -0.32) |
|  | EC | -0.12 | 0.10 | -1.21 | 0.22 | (-0.33, 0.08) |
|  | NO_2_^−^ | -0.25 | 0.10 | -2.49 | 0.01 | (-0.45, -0.05) |
| DCF rates | TOC | 0.70 | 0.06 | 11.53 | 0.00 | (0.58, 0.82) |
|  | Gene abundance | 0.22 | 0.08 | 2.81 | 0.00 | (0.07, 0.37) |
|  | Biodiversity | 0.16 | 0.07 | 2.12 | 0.03 | (0.01, 0.30) |

**References**

1. Long XE, Yao H, Wang J, Huang Y, Singh BK, Zhu YG. Community structure and soil pH determine chemoautotrophic carbon dioxide fixation in drained paddy soils. Environ Sci Technol. 2015;49:7152-60.

2. Spohn M, Muller K, Hoschen C, Mueller CW, Marhan S. Dark microbial CO_2_ fixation in temperate forest soils increases with CO_2_ concentration. Global Change Biol. 2020;26:1926-35.

3. Zhao K, Kong WD, Wang F, Long XE, Guo CY, Yue LY, et al. Desert and steppe soils exhibit lower autotrophic microbial abundance but higher atmospheric CO_2_ fixation capacity than meadow soils. Soil Biol Biochem. 2018;127:230-8.

4. Wang JC, Zou YK, Di Gioia D, Singh BK, Li QF. Conversion to agroforestry and monoculture plantation is detrimental to the soil carbon and nitrogen cycles and microbial communities of a rainforest. Soil Biol Biochem. 2020;147:107849.

5. Ge ZM, Guo HQ, Zhao B, Zhang LQ. Plant invasion impacts on the gross and net primary production of the salt marsh on eastern coast of China: Insights from leaf to ecosystem. J Geophys Res-Biogeo. 2015;120:169-86.

6. Bartoń K. MuMIn: Multi-Model Inference, R package version 1.47.5. 2023. https://cran.r-project.org/web/packages/MuMIn/.

7. Rosseel Y. lavaan: An R package for structural equation modeling. J Stat Softw. 2012;48:1-36.

8. Nanba K, King GM, Dunfield K. Analysis of facultative lithotroph distribution and diversity on volcanic deposits by use of the large subunit of ribulose 1,5-bisphosphate carboxylase/oxygenase. Appl Environ Microb. 2004;70:2245-53.

9. Alfreider A, Vogt C, Hoffmann D, Babel W. Diversity of ribulose-1,5-bisphosphate carboxylase/oxygenase large-subunit genes from groundwater and aquifer microorganisms. Microb Ecol. 2003;45:317-28.

10. Qi L, Zheng Y, Hou L, Liu B, Zhou J, An Z, et al. Potential response of dark carbon fixation to global warming in estuarine and coastal waters. Global Change Biol. 2023;29:3821-32.

11. Alfreider A, Baumer A, Bogensperger T, Posch T, Salcher MM, Summerer M. CO_2_ assimilation strategies in stratified lakes: Diversity and distribution patterns of chemolithoautotrophs. Environ Microbiol. 2017;19:2754-68.

12. Yakimov MM, La Cono V, Denaro R. A first insight into the occurrence and expression of functional *amoA* and *accA* genes of autotrophic and ammonia-oxidizing bathypelagic Crenarchaeota of Tyrrhenian Sea. Deep-Sea Res PT II. 2009;56:748-54.

13. Muyzer G, de Waal EC, Uitterlinden AG. Profiling of complex microbial populations by denaturing gradient gel electrophoresis analysis of polymerase chain reaction-amplified genes coding for 16S rRNA. Appl Environ Microb. 1993;59:695-700.

14. Cadillo-Quiroz H, Brauer S, Yashiro E, Sun C, Yavitt J, Zinder S. Vertical profiles of methanogenesis and methanogens in two contrasting acidic peatlands in central New York State, USA. Environ Microbiol. 2006;8:1428-40.
